# The effects of distractors on brightness perception based on a spiking network

**DOI:** 10.1038/s41598-023-28326-4

**Published:** 2023-01-27

**Authors:** Weisi Liu, Xinsheng Liu

**Affiliations:** 1 School of Mathematics and Information Sciences, Yantai University, Yantai, 264005 China; 2 State Key Laboratory of Mechanics and Control of Mechanical Structures, Institute of Nano Science and Department of Mathematics, Nanjing University of Aeronautics and Astronautics, Nanjing, 210016 China

**Keywords:** Neural circuits, Network models, Neural encoding

## Abstract

Visual perception can be modified by the surrounding context. Particularly, experimental observations have demonstrated that visual perception and primary visual cortical responses could be modified by properties of surrounding distractors. However, the underlying mechanism remains unclear. To simulate primary visual cortical activities in this paper, we design a k-winner-take-all (k-WTA) spiking network whose responses are generated through probabilistic inference. In simulations, images with the same target and various surrounding distractors perform as stimuli. Distractors are designed with multiple varying properties, including the luminance, the sizes and the distances to the target. Simulations for each varying property are performed with other properties fixed. Each property could modify second-layer neural responses and interactions in the network. To the same target in the designed images, the modified network responses could simulate distinguishing brightness perception consistent with experimental observations. Our model provides a possible explanation of how the surrounding distractors modify primary visual cortical responses to induce various brightness perception of the given target.

## Introduction

Brightness perception is a fundamental function of the primary visual cortex and has been explored by previous studies^1–5^. Brightness perception of a given stimulus has been demonstrated to depend on the surrounding contexts^1,6^. Particularly, it is a common situation that the surrounding context consists of distractors which could modify visual perception of the given stimulus^7–10^. However, the underlying mechanism of how the distractors modulate visual cortical responses to generate different brightness perception of the same stimulus remains unclear.

In brightness perception of the same stimulus, neural responses are modulated by contexts around the stimulus. Contextual modulation is a common phenomenon during visual perception, including brightness perception^2,6,11–16^. Perceived brightness of a stimulus is affected by the surrounding context^2,6^. A recent experimental study has suggested that illusory brightness perception known as simultaneous brightness contrast is associated with low-level visual process prior to binocular fusion^2^. In experiments with complex stimuli, brightness perception of the target is influenced strongly by perceptual organizations and considered to be relative with high-level visual system^6^. The previous study on brightness perception has found that primary visual cortical neurons accomplish spatial integration of contextual information rather than response to illumination of the retina strictly^3^. Over extracellular recordings made in the retina, the lateral geniculate nucleus and the striate cortex during experiments, neural responses in the striate cortex were found to be correlated with brightness perception under all the designed conditions^4^. Besides brightness perception, contextual modulation also occurs during other visual experiments^11,12^. Through experiments with oriented flanking stimuli, short-range connections within local neural circuitry have been found to mediate contextual modulation of neural responses to angular visual features^11^. In experiments, the varying relative contrast of stimuli in neural receptive visual fields can cause contextual effects and impact the neural stimulus-selectivity^12^. Contextual modulation also appears in visual experiments of the figure-ground segregation, the shape aftereffect, the neural sensitivity to naturalistic image and the recognitions to associated objects^13–16^. Particularly, in the surrounding context, properties of distractors have been demonstrated in experiments with influences on neural responses and visual perception^17,18^. For example, the sizes of distractors, the contrast between distractors and the stimulus, as well as the distance from distractors to the stimulus, have effects on visual perception^7–9^. Through analyses of visual neural responses and the contrast of images, Rodriguez and Granger present a generalized formulation of visual contrast energy and provide an explanatory framework for performances of identifying targets when distractors have the varying flanking distance and number^7^. Through experiments with designed flanking stimuli, Levi and Carney has found that the bigger flanks can lead to the smaller crowding phenomena^9^. Recently, with primary visual cortical neural responses of macaque monkeys to oriented stimuli, the influence of distances between distractors and the visual stimulus has been explained as a kind of spatial contextual effect on primary visual cortical activities^8^. In this way, effects of surrounding distractors on visual perception might be interpreted as a kind of contextual modulation of primary visual neural responses.

The primary visual cortex is a basic biological structure associated with a broad range of visual researches, including sparse responding, object recognition, contextual modulation and brightness perception^2,4,11–13,19–28^. The unsupervised learning within the primary visual cortex has been considered widely in neuroscience research, including the emergence of visual misperception^29–32^. In contextual modulation, both neural responses and neural interactions in the primary visual cortex could be modified^11,13,33^. Based on experimental observations, various models have been presented to explore visual perception. Batard and Bertalmío have improved a type of image processing technique which is based on differential geometry with properties of the human visual system and covariant derivatives, applying the image processing technique in exploration of brightness perception and color images correction^5^. The networks have performed as the effective models in exploring visual recognition and perception^32,34–36^. Considering interactions between boundary and feature signals in brightness perception, Pessoa, Mingolla and Neumann have developed a neural network which can implement boundary computations and provide a new interpretation of feature signals through the explicit representation of contrast-driven and luminance-driven information^35^. To simulate the visual cortex combined binocular information, Grossberg and Kelly have designed a binocular neural network which computes image contrasts in each monocular channel and fuses these discounted monocular signals with nonlinear excitatory and inhibitory signals to represent binocular brightness perception^36^. Considering influences of neural interactions, both contextual modulation and brightness perception in the visual cortex could be explored through the neural networks^37,38^. To explore the influence of distractors in brightness perception, contextual modulation of primary visual cortical neural responses and interactions might be the possible factors.

Probabilistic inference performs as the feasible exploring method for the mechanism of visual perception and neural coding^39–44^. Murray has presented a probabilistic graphical model with assumptions about lighting and reflectance to infer globally optimal interpretations of images, exhibiting partial lightness constancy, contrast, glow, and articulation effects^39^. To estimate brightness perception of images with spatial variation considered, Allred and Brainard have developed a Bayesian algorithm with the prior distributions designed to allow spatial variation in both illumination and surface reflectance and explored changes in image luminance from the aspect of spatial location^44^. Based on probabilistic inference, the winner-take-all (WTA) networks are designed for researches on visual cortical computational functions^45–48^. For simplification, these WTA networks are designed in the two-layer structure^45–47^. With neural spiking sequences generated, the plastic weights in these WTA networks have the learning rules as the Hebbian spike-timing-dependent plasticity (STDP) which is a kind of unsupervised learning^49^. With the Hebbian STDP, primary visual cortical sparse responses could be simulated from the WTA network^28,46^. The temporal structure of the neural spiking train could contribute to the information transformation compared to the rate-based neural coding^50^. The WTA networks consist of excitatory pyramidal neurons with lateral inhibitions, following neural microcircuits observed in layers 2/3^51^. Besides, the k-winner-take-all (k-WTA) network could simulate the observed simultaneous activities of multiple neurons in experiments^45^. Constructing the k-WTA spiking networks, our previous studies have explored the primary visual cortical neural responding variability and illusory stereo perception^52,53^. Yet, these WTA networks have not considered the influence of distractors on cortical neural responses and brightness perception.

In this paper, the plastic two-layer k-WTA spiking network is constructed to explore how varying properties of distractors induce contextual modulation of neural responses to generate different brightness perception of the given stimulus. Connective weights have the STDP learning rule. Afferent neurons in the first layer of the network transform the visual information into feedforward Poisson spiking trains towards the second layer. There are inter-connected excitatory and inhibitory neurons in the second-layer network. Both excitatory and inhibitory neurons in the second layer generate responses in the stochastic methods. The network recognizes outside images depending on second-layer excitatory responses. In simulations, visual images are designed with the given target stimulus and various surrounding distractors. Distractors are designed with different grayscale values, sizes and distances to the target. The influence of one varying property is explored through simulations with other properties fixed. Modulations on neural responses and lateral interactions induced by each property are measured over simulations. Brightness perception is simulated with responses from the network. Our simulations show that, to designed stimuli, varying properties of distractors could modulate second-layer neural responses and lateral interactions, inducing different brightness perception of the same target.

This article is structured as follows. In Materials and methods, the network and the corresponding probabilistic model are introduced. In the section of Results, images with various distractors perform as visual stimuli. For the designed network, neural responding modulations and brightness perception induced by distractors are investigated. With the same target stimulus and the same background, varying distractors can induce modulations of second-layer excitatory neural spiking responses and interactions, leading to different brightness perception. Finally, a conclusion is given.

## Materials and methods

### The structure of the spiking network

**Figure 1:**
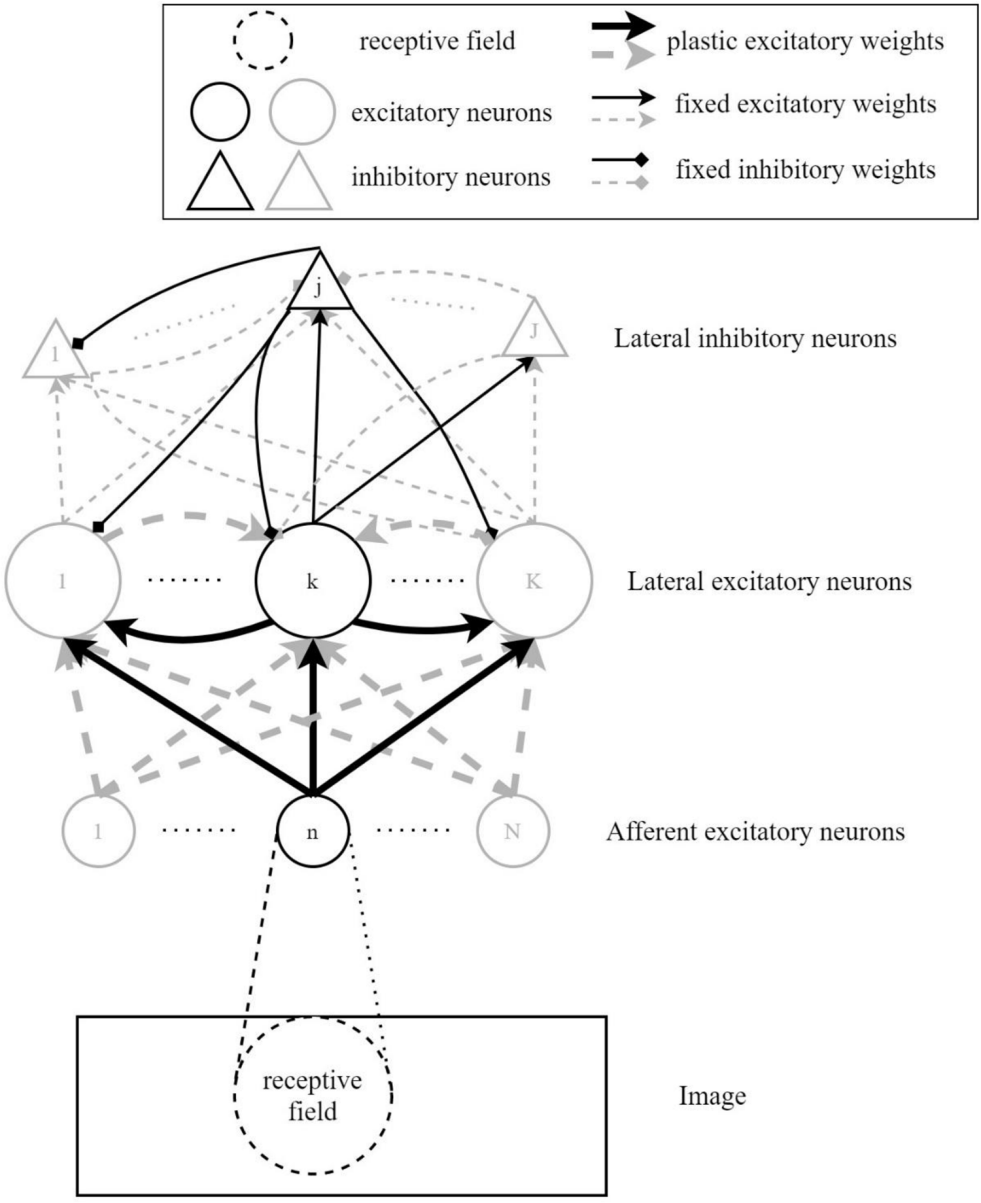
The construction of the designed k-WTA spiking network. Solid circles indicated excitatory neurons. Solid triangles indicated inhibitory neurons. The network contained first-layer afferent excitatory neurons, second-layer excitatory neurons and second-layer inhibitory neurons. Lines with arrows indicated excitatory connections. Lines with squares indicated inhibitory connections. Plastic connections were represented by thick lines. Fixed connections were represented by thin lines. The dotted circular region within the bottom image indicated the afferent neural receptive field. Afferent neurons were induced by stimuli within receptive fields to generate Poisson spikes.

In this subsection, the construction of our k-WTA network was introduced (Fig. 1). The network was designed following the basic neural circuit in the layer 2/3 for simulating primary visual cortical neural activities^45,51^. In the network, there were *N* first-layer afferent excitatory neurons, *K* second-layer excitatory neurons and *J* second-layer inhibitory neurons. In the second layer, inter-connections among neurons were designed according to specific connective probabilities in the previous experiment and the study^45,51^. With connective probabilities, the structure of the network accorded with the observed neural circuit in the layer 2/3. Influences of both neural interactions and connective plasticity in visual perception were considered in the network^54,55^.

In Fig. 1, the black circle and triangle performed as example neurons while the gray ones indicated the other neurons. The black lines presented for the synaptic connections from the example neurons while the gray lines indicated the other connections. The excitatory and inhibitory connective weights were presented by arrows and squares, respectively. The thin lines indicated the fixed weights. The plastic excitatory connective weights were expressed by the thick lines with arrows.

Each second-layer excitatory neuron received excitatory inputs from both first-layer afferent neurons and other second-layer lateral excitatory neurons. Matrices $W \in R_{K \times N}$ and $V \in R_{K \times K}$ were excitatory feedforward and lateral connective weights, respectively. Each second-layer excitatory neuron had random connections from a subset of second-layer inhibitory neurons. With $p_{EI} = 0.6$ as the special connective probability, each excitatory neuron was randomly decided to be connected by an inhibitory neuron or not. With the inputs mentioned above, the *k*th second-layer excitatory neuron had the temporal membrane potential at the $t$ th timestep as:

1 $$u_{tk}^{z} = \sum\limits_{n} {w_{kn} \tilde{x}_{tn} } + \sum\limits_{{k^{\prime } }} {v_{{kk^{\prime } }} \tilde{z}_{{tk^{\prime } }} } - \sum\limits_{{j \in J_{k} }} {v^{EI} \tilde{y}_{tj}^{{}} } + b_{k} ,$$

where $u_{tk}^{z}$ was the temporal neural membrane potential, $w_{kn} \tilde{x}_{tn}$ and $v_{{kk^{\prime } }} \tilde{z}_{{tk^{\prime } }}$ determined values of feedforward excitatory postsynaptic potentials (EPSP) induced by the $n$ th first-layer afferent neuron and the $k^{\prime}$ th second-layer excitatory neuron, respectively. $w_{kn}$ was the excitatory feedforward weight from the $n$ th neuron in the first layer. $v_{kk'}$ was the excitatory lateral weight from the $k^{\prime}$ th second-layer excitatory neuron. The feedforward weights, as well as the lateral weights between excitatory neurons in the second layer were plastic and limited in $(0,1)$. The excitability of the *k*th second-layer excitatory neuron was controlled by the parameter $b_{k}$. In our simulations, $b_{k}$ was sampled from the uniform distribution of $(0,1)$. Indices of second-layer inhibitory neurons projecting to the *k*th second-layer excitatory neuron were collected into a set as $J_{k}$. At the $t$ th timestep, the *j*th inhibitory neuron among $J_{k}$ induced the inhibitory postsynaptic potential (IPSP) as $v^{EI} \tilde{y}_{tj}$. Lateral connective weights projected to excitatory neurons from inhibitory neurons were fixed in our network and expressed as a common parameter $v^{EI}$. Because excitatory weights were limited in $(0,1)$, $v^{EI}$ was equal to the mean excitatory connective strength of 0.5 in our simulations. With neural spiking trains from corresponding neurons, $\tilde{x}_{tn}$, $\tilde{z}_{{tk^{\prime } }}$ and $\tilde{y}_{tj}^{{}}$ were the synaptic traces at the $t$ th timestep. If the *k*′th second-layer excitatory neuron generated its spikes at $\left\{ {t_{{k^{\prime}}}^{\left( 1 \right)} ,t_{{k^{\prime}}}^{\left( 2 \right)} ,t_{{k^{\prime}}}^{\left( 3 \right)} , \cdots } \right\}$, its temporal synaptic trace was given as:

2 $$\begin{aligned} \tilde{z}_{{tk^{\prime}}} & = \sum\limits_{f} {\varepsilon \left( {t - t_{{k^{\prime}}}^{\left( g \right)} } \right)} , \\ \varepsilon \left( {t - t_{{k^{\prime}}}^{\left( g \right)} } \right) & = \left\{ {\begin{array}{*{20}l} {\exp \left[ {{{ - \left( {t - t_{{k^{\prime}}}^{\left( g \right)} } \right)} \mathord{\left/ {\vphantom {{ - \left( {t - t_{{k^{\prime}}}^{\left( g \right)} } \right)} {\tau_{r} }}} \right. \kern-0pt} {\tau_{r} }}} \right] - \exp \left[ {{{ - \left( {t - t_{{k^{\prime}}}^{\left( g \right)} } \right)} \mathord{\left/ {\vphantom {{ - \left( {t - t_{{k^{\prime}}}^{\left( g \right)} } \right)} {\tau_{f} }}} \right. \kern-0pt} {\tau_{f} }}} \right],} \hfill & {if\quad t \ge t_{{k^{\prime}}}^{\left( g \right)} } \hfill \\ {0,} \hfill & {else} \hfill \\ \end{array} ,} \right. \\ \end{aligned}$$

where $\varepsilon \left( \cdot \right)$ was the synaptic response kernel, $t_{{k^{\prime}}}^{\left( g \right)}$ was the generated time of the $g$ th neural spike. The rise-time constant and the fall-time constant in the synaptic response kernel were set as $\tau_{r} = 1$ timestep, $\tau_{f} = 10$ timesteps^45^. With the common kernel $\varepsilon \left( \cdot \right)$, $\tilde{x}_{tn}$ and $\tilde{y}_{tj}^{{}}$ in Eq. (1) could be expressed similarly.

At the timestep $t$, whether a neuron generated a spike or not was presented by another variable as the temporal neural active state. In this paper, 1 ms in experiments was represented by a timestep in simulations. It was assumed in the simulations that only one spike could be generated by each neuron at most during each timestep. Under this assumption, the *k*th excitatory neural active state at $t$ was defined as a variable $z_{kt} \in \left\{ {0,1} \right\}$. Depending exponentially on the temporal neural membrane potential, the distribution of $z_{kt}$ could be expressed as:

3 $$p\left( {z_{tk} \left| {u_{tk}^{z} } \right.;\Theta } \right) = \frac{{\exp \left( {u_{tk}^{z} z_{tk} } \right)}}{{\sum\nolimits_{{z^{\prime}_{tk} \in \left\{ {0,1} \right\}}} {\exp \left( {u_{tk}^{z} z^{\prime}_{tk} } \right)} }},$$

where the neural active state was denoted as $z_{tk}$ at *t*, $u_{tk}^{z}$ was the temporal neural membrane potential in Eq. (1). For normalization, another variable $z^{\prime}_{tk}$ in the denominator took all the possible values of the neural active state. At each timestep *t*, a sample was generated from the distribution in Eq. (3) as the value of $z_{tk}$. $z_{tk} = 1$ indicated that the $k$ th excitatory neuron emitted a spike at *t*. $z_{tk} = 0$ indicated the silence of this neuron. If the $k$ th excitatory neuron generated a spike, it had a refractory period of 5 timesteps and was set to be silent^56^. Neural active states reflected neural spiking responses generated from the network.

The network could simulate identifications of stimuli at each timestep *t* through the comparisons of the vector of excitatory neural responses $\vec{z}_{t} = \left( {z_{t1} , \cdots ,z_{tK} } \right)^{\prime }$ in the second layer and the clustering sets^53^. If there were $S$ stimuli in simulations, the network identified the temporal stimulus through its temporal action $a_{t} \in \left\{ {1,2, \cdots ,S} \right\}$. With the $s$ th outside stimulus presented, the identification from the network was correct if $a_{t} = s$ or incorrect otherwise. The network could receive the temporal reward as $r_{t} = 1$ from the correct identification and $r_{t} = 0$ from the incorrect identification. From previous correct identifications to each stimulus, excitatory neural responses in the second layer were collected into the clustering set for comparisons and identifications in the next. To $S$ stimuli, clustering sets were marked as $C = \left\{ {c_{s} ,s = 1 \cdots ,S} \right\}$ with a matrix $c_{s}$ containing previous neural responses to the $s$ th stimulus. The network received temporal rewards from identifications to update both clustering sets and plastic connective weights, which was introduced in the next subsection and the supplementary material.

In the second layer, the inhibitory neural spikes were generated in the stochastic method. With the frequency-current (f-I) curve, the instantaneous firing rate of the *j*th second-layer inhibitory neuron was given as^45^:

4 $$\rho_{tj}^{y} = \sigma_{{{\text{rect}}}} \left( {u_{tj}^{y} } \right),u_{tj}^{y} = \sum\limits_{{k \in \varphi_{j} }} {v^{IE} \tilde{z}_{tk} } - \sum\limits_{{j^{\prime} \in \varsigma_{j} }} {v^{II} \tilde{y}_{{tj^{\prime}}} } ,$$

where $u_{tj}^{y}$ was the membrane potential of the *j*th lateral inhibitory neuron at *t*. $\rho_{tj}^{y}$ was the temporal neural firing rate. $\sigma_{rect} \left( \cdot \right)$ was the linear rectifying function. For $u_{tj}^{y} \ge 0$, $\sigma_{rect} \left( {u_{tj}^{y} } \right) = u_{tj}^{y}$. Otherwise, $\sigma_{rect} \left( {u_{tj}^{y} } \right) = 0$. With $p_{IE} = 0.575\left( {p_{II} = 0.55} \right)$ as the specific connective probability, each excitatory (inhibitory) neuron in the second layer was decided to have the lateral connection to the given inhibitory neuron or not. $\varphi_{j} \left( {\varsigma_{j} } \right)$ included the indices of second-layer excitatory (inhibitory) neurons those connected to the *j*th second-layer inhibitory neuron. Through stochastic decisions of second-layer lateral connections, the network could have the structure as the experimental observations^51^. Second-layer excitatory (inhibitory) neurons connected to each inhibitory neuron with the common lateral connective weight as $v^{IE} \left( {v^{II} } \right)$. Same with $v^{EI}$ in Eq. (1), $v^{IE} = 0.5\left( {v^{II} = 0.5} \right)$ in our simulations. In Eq. (4), the temporal synaptic trace of the *k*th lateral excitatory neuron (the *j′*th lateral inhibitory neuron) was expressed as $\tilde{z}_{tk}$($\tilde{y}_{{tj^{\prime}}}$). With the temporal neural firing rate $\rho_{tj}^{y}$, Poisson spikes of the *j*th second-layer inhibitory neuron were generated. The absolute refractory period of 3 timesteps was set for inhibitory neurons^45^. The temporal active state of the *j*th second-layer inhibitory neuron was expressed as $y_{tj} \in \left\{ {0,1} \right\}$.

Through Poisson spikes, the afferent neurons transmitted visual stimuli towards the second-layer network. For the visual images in simulations, the stimulus projected into the receptive field could induce afferent neural responses. Various kinds of Gaussian filters had been applied to explore brightness perception^57,58^. In this paper, the receptive field of the *n*th afferent neuron was modeled with the Difference-of-Gaussians filter^59^:

5 $$\begin{gathered} f_{n} \left( {\overrightarrow {x} ,\overrightarrow {x}_{n} } \right) = \left[ {g_{n}^{c} \left( {\overrightarrow {x} ,\overrightarrow {x}_{n} } \right) - \phi \cdot g_{n}^{s} \left( {\overrightarrow {x} ,\overrightarrow {x}_{n} } \right)} \right], \hfill \\ g_{n}^{c} \left( {\overrightarrow {x} ,\overrightarrow {x}_{n} } \right) = \exp \left\{ { - \left[ {\left( {x_{1} - x_{n1} } \right)^{2} + \left( {x_{1} - x_{n2} } \right)^{2} } \right]/\sigma_{n}^{{c^{2} }} } \right\}, \hfill \\ g_{n}^{s} \left( {\overrightarrow {x} ,\overrightarrow {x}_{n} } \right) = \exp \left\{ { - \left[ {\left( {x_{1} - x_{n1} } \right)^{2} + \left( {x_{1} - x_{n2} } \right)^{2} } \right]/\sigma_{n}^{{s^{2} }} } \right\}, \hfill \\ \end{gathered}$$

where $\overrightarrow {x} = \left( {x_{1} ,x_{2} } \right)$ was the point in the image, $\overrightarrow {x}_{n} = \left( {x_{n1} ,x_{n2} } \right)$ was the center of the $n$ th afferent receptive field. The *n*th afferent neural receptive field included center and surround Gaussian functions as $g_{n}^{c} \left( \cdot \right)$ and $g_{n}^{s} \left( \cdot \right)$. Two Gaussian functions had the spatial radii as $\sigma_{n}^{c}$ and $\sigma_{n}^{s}$. The ratio between two Gaussian functions was described as the parameter $\phi$. Parameters in DoG functions were generated from the distributions in^59^. With a spacing distance of 2 pixels, the afferent neural receptive fields were positioned on a grid to cover the image cooperatively^60^.

Visual images in our simulations were $60 \times 60$-pixel squares with different distractors which were introduced in Results in detail. Because of designed spacing distances between afferent receptive fields, there were 900 afferent neurons in the first-layer network. The second-layer network consisted of 100 excitatory neurons and 50 inhibitory neurons.

### Probabilistic model and unsupervised identifications of images

Through probabilistic inference, the network generated neural responses and identified the outside images. The probabilistic model and unsupervised identifications were similar with those in our previous study^53^. The detail introduction was presented in the supplementary material.

In simulations, the image performed as the visual stimulus $Sti$. The network simulated neural responses in the second layer through the Hidden Markov model (HMM)^46^. At the timestep *t*, the temporary observed pattern $\overrightarrow {o}_{t}$ was the instantaneous feedforward input traces $\vec{\tilde{x}}_{t} = \left( {\tilde{x}_{t1} , \cdots ,\tilde{x}_{tN} } \right)^{T}$. Second-layer neural responses $\left\{ {\vec{z}_{t} ,\vec{y}_{t} } \right\}$ and lateral synaptic traces $\left\{ {\vec{\tilde{z}}_{t} ,\vec{\tilde{y}}_{t} } \right\}$ were included in the temporal hidden state $\vec{h}_{t}$. Plastic parameters in the model were collected as $\Theta = \left\{ {W,V,C} \right\}$. $W \in R_{K \times N}$ and $V \in R_{K \times K}$were matrices of plastic connections. Clustering sets to stimuli were presented as $C = \left\{ {c_{s} ,s = 1 \cdots ,S} \right\}$.

For the timestep $t$, $\vec{O}_{t} = \left( {\vec{o}_{1} ,\vec{o}_{2} , \cdots ,\vec{o}_{t} } \right)$ included occurred observations and $\vec{H}_{t - 1} = \left( {\vec{h}_{1} ,\vec{h}_{2} , \cdots ,\vec{h}_{t - 1} } \right)$ presented hidden states up to the timestep $t - 1$. The stochastic dynamics of the k-WTA network implemented a forward sampling process and sampled a new hidden active state $\vec{h}_{t}$ forward in time based on $\vec{O}_{t}$ and $\vec{H}_{t - 1}$ ^46^. In the sampling of $\vec{h}_{t}$, excitatory and inhibitory neural responses in the second layer were generated by Eqs. (3) and (4), which were introduced in detail in our previous study^52^. At each timestep, the network generated an action $a_{t}$ to identify stimuli with second-layer excitatory neural spikes and obtained a temporal reward $r_{t} \in \left\{ {0,1} \right\}$.

Biologically, the primary visual cortical neural responses are modified by dopaminergic rewards^61^. The previous WTA network had considered the reward-modified Hebbian learning^62^. In this paper, modulations of plastic weights at each timestep depended on the temporal reward $r_{t}$. $r_{t} = 1$ at the timestep $t$ indicated that the network made a correct identification. Then, plastic weights were updated at $t$ according to generated neural responses. Up to $t$, sequences of observed variables and hidden variables were marked as $\vec{O}_{t} = \left( {\vec{o}_{1} ,\vec{o}_{2} , \cdots ,\vec{o}_{t} } \right)$, $\vec{H}_{t} = \left( {\vec{h}_{1} ,\vec{h}_{2} , \cdots ,\vec{h}_{t} } \right)$. For $r_{t} = 1$, the connective modulations are impacted by several sub-sequences of dynamics with their lengths as $T \in \left\{ {1, \cdots ,t} \right\}$. Up to $t$, the $T$-step sub-sequences of observed variables and hidden variables were marked as $\vec{O}_{T} = \left( {\vec{o}_{t - T + 1} , \cdots ,\vec{o}_{t} } \right),\vec{H}_{T} = \left( {\vec{h}_{t - T + 1} , \cdots ,\vec{h}_{t} } \right)$. With the discount factor $\gamma \in \left( {0,1} \right)$, the contribution of this pair of $T$- step sub-sequences to $r_{t}$ was given as $\alpha \left( T \right) = \left( {1 - \gamma } \right)\gamma^{T - 1}$. With all the sub-sequences of neural dynamics considered, the likelihood function $L\left( \Theta \right)$ was expressed as^62^:

6 $$L\left( \Theta \right) = \sum\limits_{\tau = 1}^{t} {\left[ {\alpha \left( \tau \right) \cdot \left\langle {r_{t} \cdot \log p\left( {\vec{O}_{\tau } ,\vec{H}_{\tau } ,Sti;\Theta } \right)} \right\rangle_{{p\left( {\vec{O}_{\tau } ,\vec{H}_{\tau } ,Sti,r_{t} } \right)}} } \right]} .$$

The joint distributions $p\left( {\vec{O}_{\tau } ,\vec{H}_{\tau } ,Sti,r_{t} } \right)$ and $p\left( {\vec{O}_{\tau } ,\vec{H}_{\tau } ,Sti;\Theta } \right)$ could be factorized with the assumption of Hidden Markov model:

7 $$\begin{aligned} p\left( {\vec{O}_{\tau } ,\vec{H}_{\tau } ,Sti,r_{t} } \right) = &\,p\left( {r_{t} \left| {\vec{H}_{\tau } } \right.} \right) \cdot p\left( {\vec{H}_{\tau } \left| {\vec{O}_{\tau } } \right.} \right) \cdot p\left( {\vec{O}_{\tau } \left| {Sti} \right.} \right) \cdot p\left( {Sti} \right), \\ p\left( {\vec{O}_{\tau } ,\vec{H}_{\tau } ,Sti;\Theta } \right)  = & \,p\left( {Sti\left| {\vec{O}_{\tau } ,\vec{H}_{\tau } ;\Theta } \right.} \right)p\left( {\vec{O}_{\tau } ,\vec{H}_{\tau } ;\Theta } \right) \\ = & \,p\left( {Sti\left| {\vec{O}_{\tau } ,\vec{H}_{\tau } ;\Theta } \right.} \right) \cdot \prod\nolimits_{t^{\prime} = t - \tau + 1}^{t} {p\left( {\vec{o}_{{t^{\prime}}} \left| {\vec{h}_{{t^{\prime}}} ;\Theta } \right.} \right)p\left( {\vec{h}_{{t^{\prime}}} \left| {\vec{h}_{{t^{\prime} - 1}} ;\Theta } \right.} \right)} , \\ p\left( {\vec{O}_{\tau} ,\vec{H}_{\tau} ;\Theta } \right) = & \sum\limits_{t^{\prime} = t - \tau + 1}^{t} {p\left( {\vec{o}_{{t^{\prime}}} \left| {\vec{h}_{{t^{\prime}}} ;\Theta } \right.} \right)p\left( {\vec{h}_{{t^{\prime}}} \left| {\vec{h}_{{t^{\prime} - 1}} ;\Theta } \right.} \right)}. \\ \end{aligned}$$

The likelihood function $L\left( \Theta \right)$ had the equivalent representation with the factorization in Eq. (7) as:

8 $$\begin{aligned} L\left( \Theta \right) = & \sum\limits_{\tau = 1}^{t} {\left\{ {\alpha \left( \tau \right) \cdot \left\langle {r_{t} \cdot \log \left[ {p\left( {Sti\left| {\vec{O}_{\tau } ,\vec{H}_{\tau } ;\Theta } \right.} \right) \cdot \prod\nolimits_{t^{\prime} = t - \tau + 1}^{t} {p\left( {\vec{o}_{{t^{\prime}}} \left| {\vec{h}_{{t^{\prime}}} ;\Theta } \right.} \right)p\left( {\vec{h}_{{t^{\prime}}} \left| {\vec{h}_{{t^{\prime} - 1}} ;\Theta } \right.} \right)} } \right]} \right\rangle_{{p\left( {\vec{O}_{\tau } ,\vec{H}_{\tau } ,Sti,r_{t} } \right)}} } \right\}} \\ = & \sum\limits_{\tau = 1}^{t} {\alpha \left( \tau \right) \cdot \left\langle {r_{t} \cdot \log p\left( {Sti\left| {\vec{O}_{\tau } ,\vec{H}_{\tau } ;\Theta } \right.} \right) + r_{t} \cdot \sum\nolimits_{t^{\prime} = t - \tau + 1}^{t} {\log \left[ {p\left( {\vec{o}_{{t^{\prime}}} \left| {\vec{h}_{{t^{\prime}}} ;\Theta } \right.} \right)p\left( {\vec{h}_{{t^{\prime}}} \left| {\vec{h}_{{t^{\prime} - 1}} ;\Theta } \right.} \right)} \right]} } \right\rangle_{{p\left( {\vec{O}_{\tau } ,\vec{H}_{\tau } ,Sti,r_{t} } \right)}} } . \\ \end{aligned}$$

For the likelihood function $L\left( \Theta \right)$ in Eq. (8), the network updated plastic connective weights through an online variant of the Expectation–Maximization algorithm. In this paper, the E-step was to estimate the expectation with a single sample of neural responses^46^. With the single sample from $p\left( {\vec{O}_{\tau } ,\vec{H}_{\tau } ,Sti,r_{t} } \right)$, $L\left( \Theta \right)$ was rearranged and approximated as:

9 $$L\left( \Theta \right) \simeq \sum\limits_{\tau = 1}^{t} {\alpha \left( \tau \right) \cdot r_{t} \cdot \left[ {\log p\left( {Sti\left| {\vec{O}_{\tau } ,\vec{H}_{\tau } ;\Theta } \right.} \right) + \sum\limits_{{t^{\prime} = t - \tau + 1}}^{t} {\log p\left( {\vec{o}_{{t^{\prime}}} \left| {\vec{h}_{{t^{\prime}}} ;\Theta } \right.} \right)p\left( {\vec{h}_{{t^{\prime}}} \left| {\vec{h}_{{t^{\prime} - 1}} ;\Theta } \right.} \right)} } \right]} .$$

Then, with directions given by partial derivatives of $L\left( \Theta \right)$, the network updated its plastic weights in the M-step^63^. In this paper, the directions for updating $w_{kn}$ and $v_{kk\prime }$ were given as^52,53^:

10 $$\begin{gathered} \frac{\partial }{{\partial w_{kn} }}L\left( \Theta \right) \simeq r_{t} \sum\limits_{{t^{\prime} = 1}}^{t} {\gamma^{{t - t^{\prime}}} \cdot z_{{t^{\prime}k}}^{{}} \cdot \left\{ {\tilde{x}_{{t^{\prime}n}}^{{}} - \left[ {1 - \frac{1}{{w_{kn} }} + \frac{1}{{\exp \left( {w_{kn} } \right) - 1}}} \right]} \right\}} , \hfill \\ \frac{\partial }{{\partial v_{kk^{\prime}} }}L\left( \Theta \right) \simeq r_{t} \sum\limits_{{t^{\prime} = 1}}^{t} {\gamma^{{t - t^{\prime}}} \cdot z_{{t^{\prime}k}} \cdot \left\{ {\tilde{z}_{{t^{\prime}k^{\prime}}} - \left[ {1 - \frac{1}{{v_{kk^{\prime}} }} + \frac{1}{{\exp \left( {v_{kk^{\prime}} } \right) - 1}}} \right]} \right\}} . \hfill \\ \end{gathered}$$

The modification of each weight depended on the pre- and postsynaptic responses, as well as the temporal reward $r_{t}$. $r_{t}$ controlled connective modifications. Connective weights only would be updated with the correct identification. In simulations, the discount factor $\gamma = 0.9$.

To identify the outside stimuli, the network applied the unsupervised method^64^. The detail generations of $a_{t}$ and $r_{t}$ were introduced in our previous study^53^. The brief description of identifications was introduced in this subsection. Previous neural responses had been identified and collected into clustering sets for latter identifications. For temporal second-layer excitatory neural spikes $\vec{z}_{t}$ and $\alpha$, we calculated energy distances between $\vec{z}_{t}$ and clustering sets $\left\{ {c_{s} ,s = 1, \cdots ,S} \right\}$ to estimate parameters $\left\{ {e_{accept} \left( {\alpha ,c_{s} ,t} \right),s = 1, \cdots ,S} \right\}$ through $R$ re-samples^64^. For $\left\{ {c_{s} ,s = 1, \cdots ,S} \right\}$, a common parameter was set as $e_{accept} (\alpha ,t) = \min \left\{ {e_{accept} \left( {\alpha ,c_{s} ,t} \right),s = 1, \cdots ,S} \right\}$. The likelihoods between $\vec{z}_{t}$ and clustering sets could be quantified by the probabilities $\left\{ {p\left( {\alpha ,c_{s} ,t} \right) = p\left( {e\left( {\alpha ,c_{s} ,t} \right) \le e_{accept} \left( {\alpha ,t} \right)} \right),s = 1, \cdots ,S} \right\}$.

To the outside stimulus, $a_{t}$ at the timestep $t$ performed as the temporal identification. The distribution of $a_{t}$ was given as:

11 $$p\left( {a_{t} = s^{\prime}} \right) = \exp \left( {p\left( {\alpha ,c_{{s^{\prime}}} ,t} \right)} \right)/\left[ {\sum\limits_{s}^{{}} {\exp \left( {p\left( {\alpha ,c_{s} ,t} \right)} \right)} } \right].$$

If $a_{t}$ took a value equal to the serial number of the presented stimulus, the temporal identification was correct. In a First-In-First-Out (FIFO) manner, the clustering set would be updated at a timestep with the correct identification. In each update, $\vec{z}_{t}$ would be added to the end of the corresponding clustering set. After updating, the clustering set would delete redundant components from its beginning if its size exceeds $n_{cluster}$. Detail introductions of updating clustering sets were introduced in our previous study^53^. In our simulations, $n_{cluster} = 20$, $\alpha = 0.05$, $R = 50$.

## Results

Similar with stimuli in the previous study^7^, gray square images with different contexts are designed in simulations. This section is to explore how distractors modify brightness perception of the target stimulus.

### The phenomenon of simultaneous brightness contrast

This subsection is to test whether our network can simulate distinguishing brightness perception of the same stimulus upon opposite backgrounds. In simulations, two sets of images are designed as visual stimuli.

**Figure 2:**
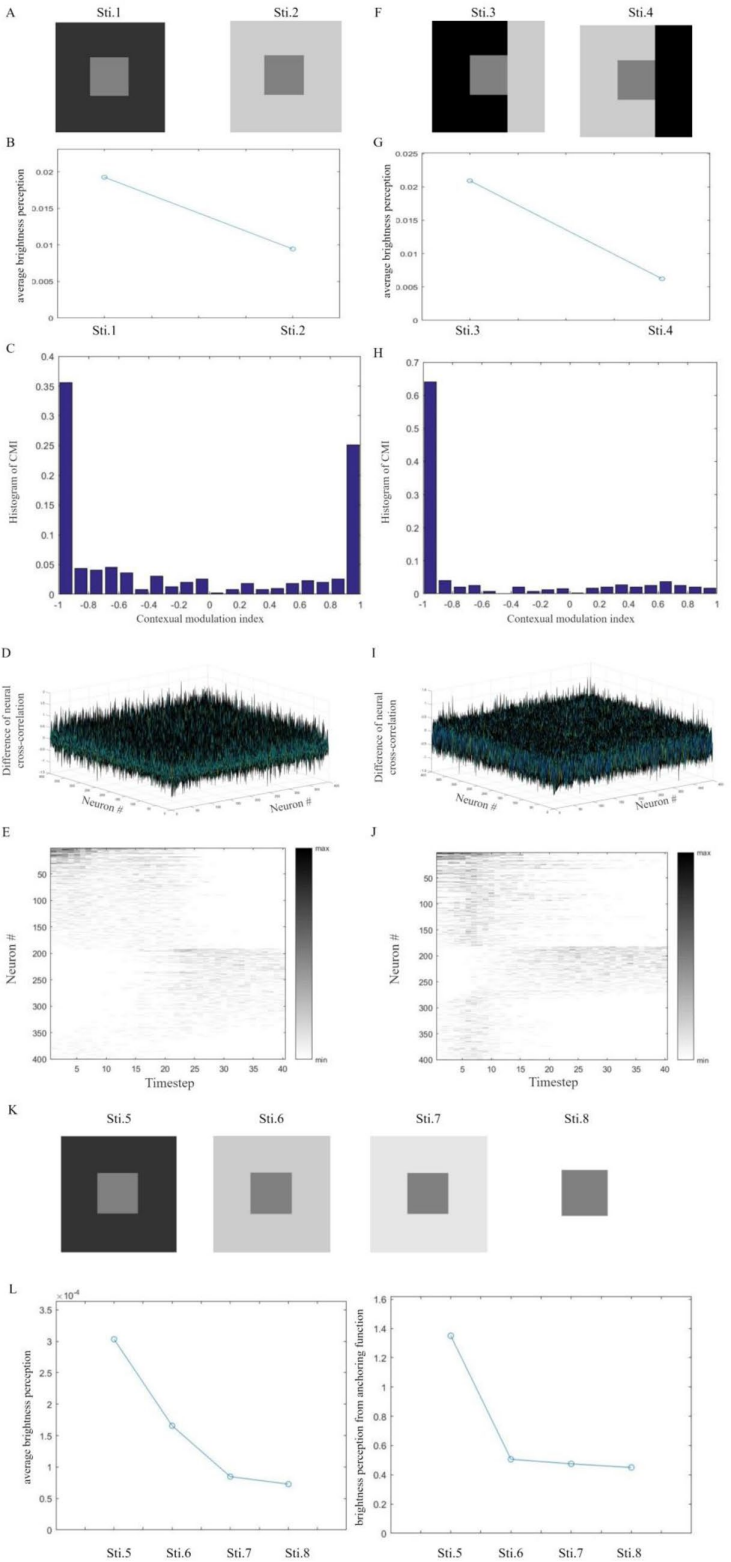
Perception of simultaneous brightness contrast from the network. (**A**) Images used as the first set of visual stimuli in simulations. (**B**) Averaged brightness perception of target squares with neural responses from our network. Perception is averaged over simulations, timesteps and pixels. Perception of the same gray square is lighter upon the darker background. (**C**) The histogram of contextual modulation indices (CMIs) about excitatory neural responses to Sti. 1 and Sti. 2. A non-zero CMI indicates contextual modulation of neural responses. (**D**) Difference of excitatory neural cross-correlations to Sti. 1 and Sti. 2. A non-zero difference indicates contextual modulation of interactions between paired neurons. (**E**) Temporal excitatory neural firing rates to Sti. 1 and Sti. 2 averaged over simulations. Color of each block indicates neural responding strength. Different images can induce intense responses of different subset of neurons. (**F**) Images used as the second set of visual stimuli in simulations. (**G**) Averaged brightness perception of target squares. (**H**) The histogram of CMIs about excitatory neural responses to Sti. 3 and Sti. 4. (**I**) Difference of second-layer excitatory neural cross-correlations to Sti. 3 and Sti. 4. (**J**) Temporal second-layer excitatory neural firing rate to Sti. 3 and Sti. 4 averaged over simulations. (**K**) Images used as the third set of visual stimuli. (**L**) Averaged brightness perception of the target from both the neural network and the anchoring function. With the background becoming lighter, varying trends of estimated brightness perception in subplots are similar.

In the first set, two images are designed by combining a grey square with darker and lighter backgrounds, respectively^65^. Each image is a $60 \times 60$-pixel square. The target stimulus is a $20 \times 20$-pixel square in the center of each image (Fig. 2A). In the second set, $60 \times 60$-pixel square images have the combined backgrounds. A $20 \times 20$-pixel target stimulus belongs to different parts in images (Fig. 2F). In both two sets of images, the grey target stimulus has a grayscale value of 0.5, the darker background has a grayscale value of 0.2 and the lighter background has a grayscale value of 0.8.

For stimuli in Fig. 2A, visual images are encoded by afferent neural spikes. In each learning simulation, two images are rearranged into a random sequence. In 200 learning simulations, connective weights and clustering sets are updated as introduced in Materials and Methods. Initial values of plastic feedforward and lateral connective weights are sampled independently from a uniform distribution of (0.001, 1). During modifications, weights are limited in (0, 1). The testing phase consists of 100 simulations with weights and clustering sets fixed. A sequence of images is presented as in Fig. 2A. Neural responses over simulations are measured to explore contextual modulation induced by distractors. For images in Fig. 2F, our network is trained and tested in the same way. Besides, learning simulations and testing simulations in all the following subsections are designed similarly.

Brightness perception of the target is simulated through reconstructions of images. Over testing simulations, a point $(x_{1} ,x_{2} )$ in each image is reconstructed with averaged neural responses as:

12 $$\begin{gathered} I_{rec}^{{}} (x_{1} ,x_{2} )\overline{ = }\sum\limits_{k,n} {\overline{z}_{k} \cdot w_{kn} \cdot \overline{y}_{n} \cdot I_{n}^{rec} (x_{1} ,x_{2} )} \hfill \\ I_{n}^{rec} (x_{1} ,x_{2} ) = a_{n}^{rec} \cdot f_{n} \left( {\vec{x},\vec{x}_{n} } \right) \hfill \\ a_{n}^{rec} = \sum\limits_{{(x^{\prime}_{1} ,x^{\prime}_{2} )}} {I\left( {x^{\prime}_{1} ,x^{\prime}_{2} } \right) \cdot f_{n} \left( {\vec{x}^{\prime},\vec{x}_{n} } \right)} , \hfill \\ \end{gathered}$$

where $\overrightarrow {x} = \left( {x_{1} ,x_{2} } \right)$ is a point in the image, $f_{n} \left( \cdot \right)$ is the Difference-of-Gaussian filter in Eq. (5) with its center as $\overrightarrow {x}_{n} = \left( {x_{n1} ,x_{n2} } \right)$. $w_{kn}$ is the modified feedforward weight. $\overline{z}_{k} ,\overline{y}_{n}$ are averaged spiking rates of corresponding neurons over timesteps and simulations. $I\left( {x^{\prime}_{1} ,x^{\prime}_{2} } \right)$ is the gray-scale value of a point $\vec{x}^{\prime} = \left( {x^{\prime}_{1} ,x^{\prime}_{2} } \right)$. $I_{rec} (x_{1} ,x_{2} )$ is the reconstructed gray-scale value of $(x_{1} ,x_{2} )$. The reconstruction in Eq. (12) performs as averaged brightness perception. Reconstructions of two images are normalized before comparison. Reconstructed gray-scale values of all the points in two images are collected together into a set. The mean value and standard deviation of this set are common parameters for normalizations. In this way, perception of the same target upon different contexts can be simulated and compared. For the first set, the target in Sti. 1 appears to be lighter (Fig. 2B). For the second set, the target in Sti. 4 appears to be lighter (Fig. 2G). Simulated brightness perception has the relationship as same as that in experiments^65^.

The contextual modulation index (CMI) and the cross-correlation are used to qualify contextual modifications of excitatory neural responses and excitatory neural lateral interactions, respectively. For instance, the CMI of the $k$ th second-layer excitatory neuron to Sti. 1 and Sti. 2 is calculated as^37^:

13 $$CMI_{k} = \frac{{r_{k1} - r_{k2} }}{{r_{k1} + r_{k2} }},$$

where $r_{k1}$ and $r_{k2}$ are spiking counts of the $k$ th excitatory neuron to Sti. 1 and Sti. 2, respectively. If $r_{k1} = r_{k2}$, responses of this neuron to two stimuli are same and $CMI_{k} = 0$. If $r_{k1} \ne r_{k2}$, two stimuli induce different responses of this neuron and $CMI_{k} \ne 0$. In this way, a non-zero CMI indicates contextual modulation of neural responses. CMI in Eq. (13) is limited within $[ - 1,1]$ by the denominator. With an absolute value closer to 1, a CMI reflects larger contextual modulation induced by different stimuli. For a neural population, contextual modulation of neural responses can be reflected by the histogram of CMIs. For second-layer excitatory neurons, non-zero CMIs in Fig. 2C,H indicate that our network can simulate contextual modulation of neural responses induced by different backgrounds.

The cross-correlation of each pair of excitatory neurons in the second layer quantify their interactions^55^. Cross-correlations are calculated with the time lag of 0. To reflect influences on neural responding cross-correlations, differences between neural responding cross-correlations to each set of images are calculated. For a pair of excitatory neurons, a non-zero difference of neural cross-correlation indicates the context can induce the modulation on their interactions. As shown in Fig. 2D,I, our network can simulate contextual modulation of neural interactions.

Our spiking network can simulate sparse neural responses to images^45,46^. To two sets of designed images in Fig. 2A,F, temporal averaged firing rates of second-layer excitatory neurons to images are calculated over testing simulations. As shown in Fig. 2E,J, neural responding strengths are expressed by the varying color. For the convenient observation, the sequence of neurons is re-arranged according to their responding strengths to each set of images. At each timestep, the darker rectangular block indicates the stronger responding strength of a neuron. The white blocks stand for the minimum responding strength of 0. To an image, some excitatory neurons emit spikes intensely while other excitatory neurons generate few spikes. Different images can induce intense neural responses of different subsets of second-layer excitatory neurons. After learning, our network can simulate sparse and distinguishing responses to different images.

We also provide brief simulations of anchoring^66^. Images in Fig. 2K perform as another set of visual stimuli. The grey target stimulus has a grayscale value of 0.5. The darker background has a grayscale value of 0.2. Other lighter backgrounds have grayscale values of 0.8, 0.9 and 1, respectively. Brightness perception simulated from the network and the anchoring function in the previous study^66^ are shown in Fig. 2L. With the background becoming lighter, varying trends of brightness perception in subplots are similar. Our network can provide simulations of the basic phenomenon of anchoring.

Simulations in this subsection show that our network can reflect contextual modulation of neural responses and interactions, simulating opposite brightness perception of the same target.

### Modulations and perception induced by distances between distractors and targets

Recent study has explored illusory brightness perception induced by different orders of presentations of two stimuli^67^. In this paper, the additional gray-scale squares are designed around the target and perform as distractors. It is to explore how distractors modify brightness perception of the given target.

**Figure 3:**
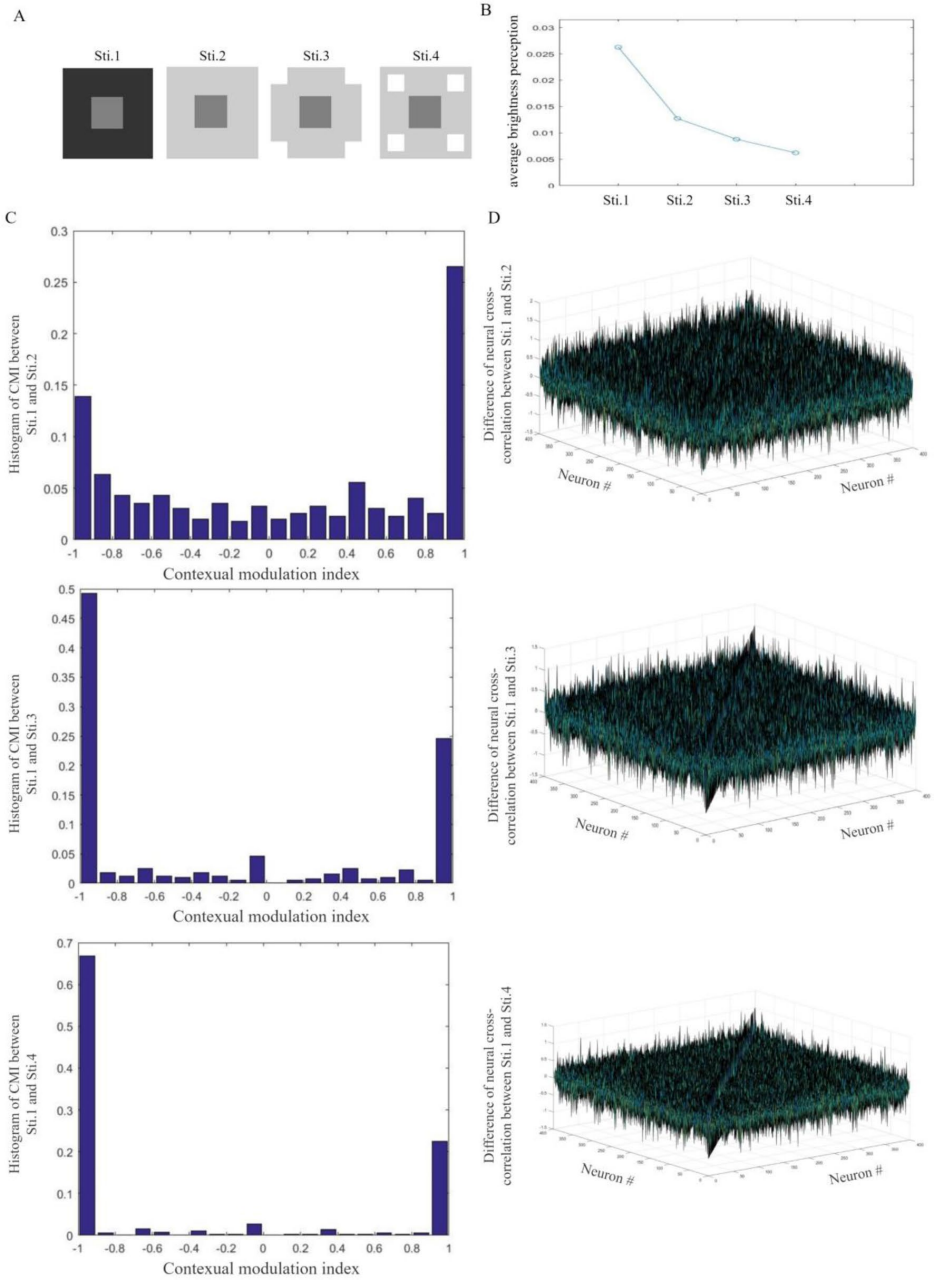
Brightness perception induced by distances between distractors and targets. (**A**) Images used as visual stimuli in our simulations. (**B**) Averaged brightness perception of target squares. For designed stimuli, distractors with a shorter distance induce darker brightness perception of the target. (**C**) The histogram of excitatory neural CMIs to stimuli. A non-zero CMI indicates the neural responding modification induced by the varying distance between distractors and the target. For a neural population, the histogram of CMIs reflects contextual modulation of neural responses. For designed stimuli, distractors with a shorter distance can induce larger neural responding modulations. (**D**) Difference of excitatory neural responding cross-correlation to stimuli. A non-zero difference indicates the modulation of neural interactions induced by the varying distance. With simulations of Sti. 1 for comparison, different amplitudes of neural interactive modification reflect influences of the varying distance.

In this subsection, distractors are designed to have different distances to targets in visual images. In our simulations, the gray patch is upon different contexts in Fig. 3A. Each image is a $60 \times 60$-pixel square. The $20 \times 20$-pixel grey target has a grayscale value of 0.5, the darker background has a grayscale value of 0.2 and the lighter background has a grayscale value of 0.8. $10 \times 10$-pixel distractors have a common grayscale value of 1. Distractors in Sti. 3 locate along boundaries of the image. Distractors in Sti. 4 become closer to the target with both horizontal and vertical moving steps of 5 pixels.

With images in Fig. 3A, 200 learning simulations and 100 testing simulations are designed for our network. Over testing simulations, a reconstruction of an image is given by Eq. (12). Average perception of the target is calculated and shown in Fig. 3B. Brightness perception of the target stimulus is darker when the distance from distractors to the target is shorter. The varying perception can reflect influences of distances between distractors and the target. The contextual modulation index (CMI) and the cross-correlations are used to qualify influences on the excitatory neural responses and lateral interactions, respectively. To calculate modulations induced by different distances, neural responses to Sti. 1 are collected for comparison. Non-zero CMIs in histograms reflect induced responding modifications of second-layer excitatory neurons (Fig. 3C). When distractors become closer to the target, there are more CMIs having the absolute value close to 1. It indicates that, for designed images, a shorter distance between distractors and the target can induce larger neural responding modulations.

Differences between neural responding cross-correlations to Sti. 1 and other stimuli are calculated. As shown in Fig. 3D, a non-zero difference of neural cross-correlation indicates the influence of the varying distance on neural interactions. With simulations of Sti. 1 for comparison, different amplitudes of neural interactive modification reflect influences of distances between distractors and the target.

Simulations show that our network can reflect contextual modification induced by distances between distractors and the target. Modified neural activities simulate different perception of the same target. For designed images in Fig. 3A a shorter distance makes our network simulate darker perception of the same target.

### Modulations and perception induced by grayscale values of distractors

**Figure 4:**
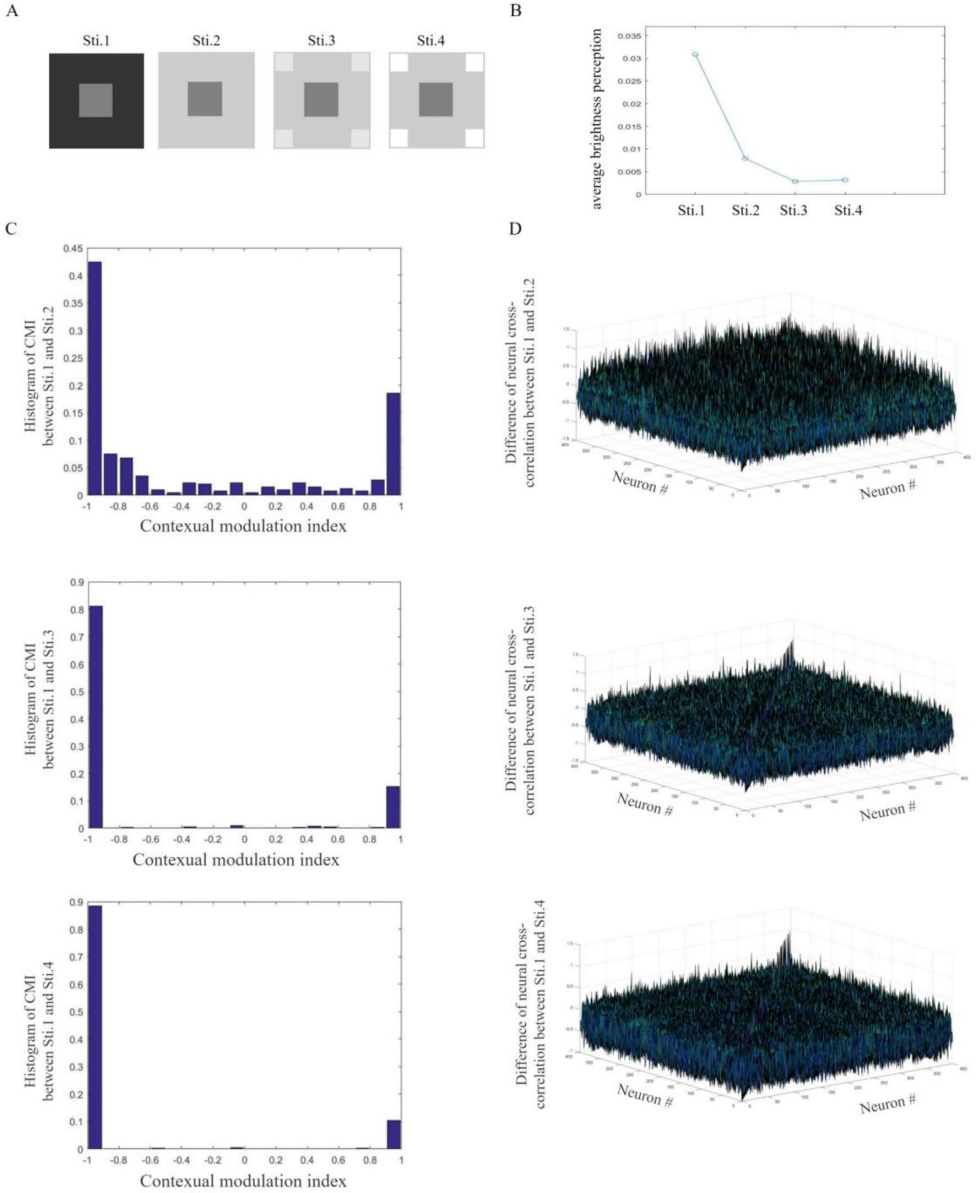
Brightness perception induced by grayscale values of distractors. (**A**) Images used as visual stimuli in our simulations. (**B**) Averaged brightness perception of target squares. For designed stimuli, the target with lighter distractors appears to be darker. (**C**) The histogram of CMIs of excitatory neurons to images. Non-zero CMIs indicate neural responding modifications induced by grayscale values of distractors. For designed stimuli, lighter distractors can induce larger neural responding modulations. (**D**) Differences of excitatory neural responding cross-correlation to stimuli. For each pair of neurons, amplitudes of non-zero differences reflect influences of the varying grayscale value of distractors.

In this subsection, distractors are designed to have different grayscale values. Four $60 \times 60$-pixel images are designed as in Fig. 4A. The target and backgrounds are the same with those in Fig. 3A. The $10 \times 10$-pixel distractors in Sti. 3 has a grayscale value of 0.9. The distractors in Sti. 4 has a grayscale value of 1.

200 learning simulations and 100 testing simulations are designed for the network. Over testing simulations, brightness perception of the target is simulated and shown in Fig. 4B. Our simulations show that the target with lighter distractors appears to be darker. With Sti. 1 for comparison, CMIs modulated by other 3 stimuli are measured for each neuron. Induced by different distractors, neural responding modulations are shown in Fig. 4C. Simulations show that, to designed images in Fig. 4A, lighter distractors can induce larger neural responding modulations. The network can reflect modifications of excitatory neural responses induced by grayscale values of distractors. Differences of neural responding cross-correlations are calculated and shown in Fig. 4D. A non-zero difference of neural cross-correlation shows how grayscale values of distractors affect neural interactions. Compared to simulations of Sti. 1, interactive modulations between each pair of neurons are induced by stimuli and reflected by amplitudes of non-zero differences.

This subsection explores how grayscale values of distractors modify the neural activities of the network. With modulated responses, our network can simulate distinguishing brightness perception induced by grayscale values of distractors. For designed images in Fig. 4A, lighter distractors make our network simulate darker perception of the target.

### Modulations and perception induced by sizes of distractors

**Figure 5:**
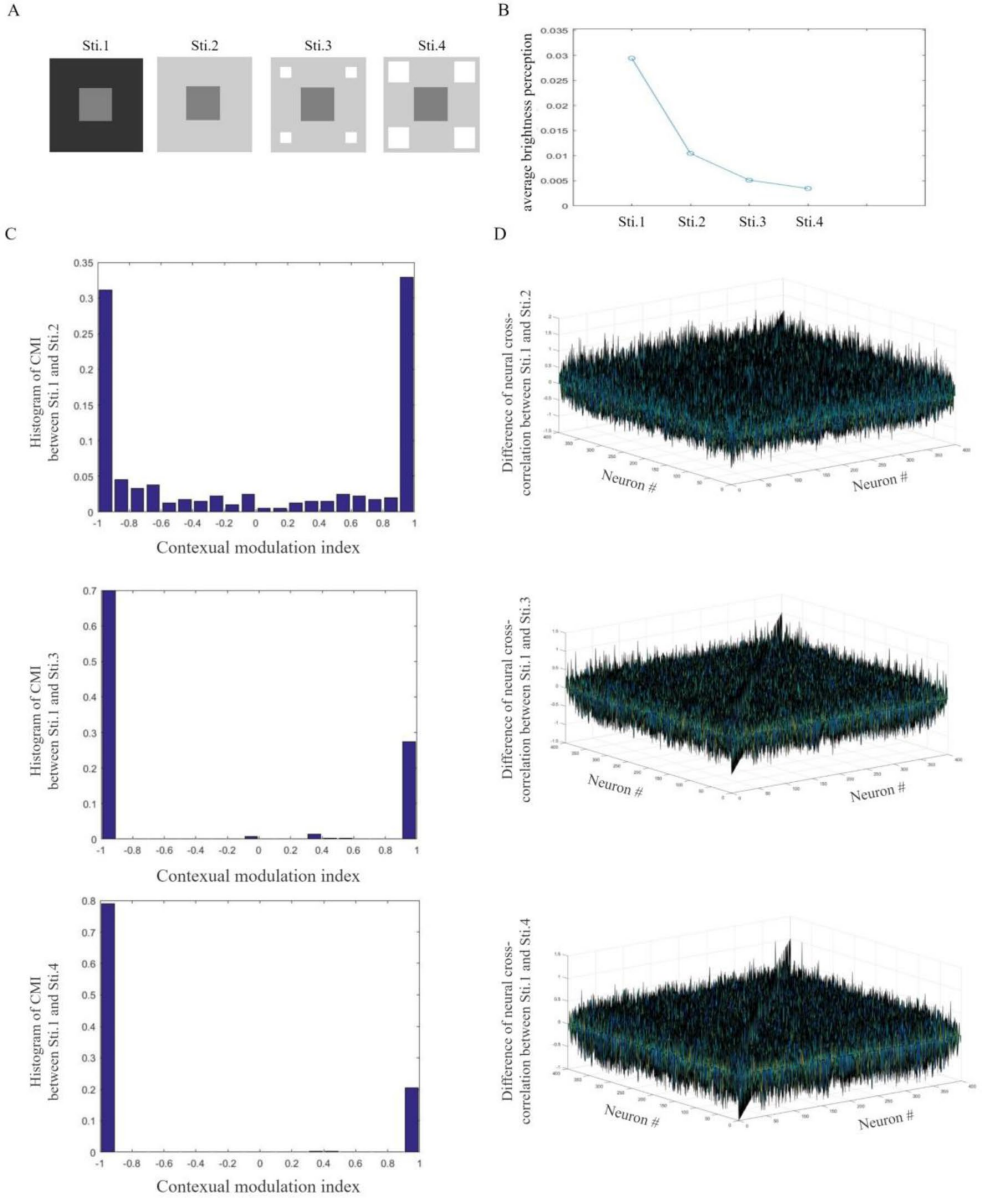
Brightness perception induced by sizes of distractors. (**A**) Images used as visual stimuli in our simulations. (**B**) Averaged brightness perception of target squares. For designed stimuli, perception of the same gray square is darker with bigger distractors. (**C**) The histogram of CMIs to stimuli. For designed stimuli, larger distractors could induce larger neural responding modulations. (**D**) Differences of excitatory neural responding cross-correlation to stimuli. A non-zero difference indicates the modulation of neural interactions induced by the varying size of distractors.

In this subsection, distractors are designed to have different sizes. The $60 \times 60$-pixel images in Fig. 5A have the same target and backgrounds as those in Fig. 3A. With a grayscale value of 1, square distractors have different sizes. In our simulations, Sti. 3 has $5 \times 5$-pixel distractors while Sti. 4 has $10 \times 10$-pixel distractors.

With stimuli in Fig. 5A, the learning phase and the testing phase are designed. Over testing simulations, reconstructions of the target given by Eq. (12) perform as brightness perception. As shown in Fig. 5B, for designed images in simulations, the stimulus with bigger distractors appears to be darker. With Sti. 1 for comparison, CMIs and differences of neural responding cross-correlations are measured for other three stimuli. The varying size of distractors could induce neural responding modulations (Fig. 5C). Distractors with larger size can lead to larger modulations of neural responses. A non-zero difference of neural cross-correlation indicates contextual modulation of neural interactions induced by the varying size (Fig. 5D).

Neural responses and interactions modified by sizes of distractors are measured and reflected from the network. With modulated responses, our network can simulate distinguishing brightness perception induced by the varying size. For designed images in Fig. 5A, bigger distractors make our network simulate darker perception of the target.

## Conclusion

This paper has provided a probabilistic framework to explore how distractors modify primary visual cortical responses and induce different brightness perception of the same stimulus. Recent experimental study has demonstrated that the phenomenon of simultaneous brightness contrast is associated with primary visual cortical neural responses^2^. Besides, contextual effects are also associated with the primary visual cortex^13,33,38^. While brightness perception has been studied for a long time, how distractors modify primary visual cortical neural responses to induce different brightness perception of the same stimulus is still not clear. To explore the corresponding mechanism, we design a stochastic spiking network with plastic connections in this study. Visual images are designed to control varying properties of distractors, excluding undesired factors in simulations.

Our network is constructed with two layers. With neural receptive fields as Difference-of-Gaussians filters, first-layer afferent neurons generate Poisson spiking responses to received stimuli. Through forward sampling in the Hidden Markov Model, second-layer excitatory and inhibitory neurons generate their spiking responses and communicate with each other. With multi-dimensional excitatory neural spiking responses in the second layer, the network identifies the presented stimulus and receives rewards which control connective modulations.

Applications of afferent receptive fields in this paper remove the strict limitation on the number of neurons in previous WTA networks while simulating sparse neural responses^45,46^. Compared to neural populational coding with Gaussian functions^58^, this model considers neural interactions and synaptic plasticity in the primary visual cortex. In this way, besides neural responses, the plasticity-based influence is also considered to explore illusory brightness perception induced by distractors^54^. Compared to the feedforward network model for visual perception with distractors, our recurrent network considers influences of neural lateral interactions^68^. In biology, the dopaminergic reward has been found to participate in the synaptic plasticity in the primary visual cortex^61^. Compared to previous networks on brightness perception^5,35,36,57^, this model provides unsupervised identifications of stimuli and considers influences of rewards on learning. This unsupervised method only depends on generated neural spikes which are easy to obtain from simulations. Without limiting the dimension of neural responses, the unsupervised identification can provide online rewards to control connective modifications.

This paper explores how properties of distractors modulate neural responses and induce different brightness perception of the given target. For images as visual stimuli in simulations, distractors are designed with the varying grayscale value, the size and the distance to the target. Over simulations, both brightness perception of the target stimulus and neural responding modulations are measured. Our network can reflect modulations on both neural responses and interactions induced by each property of distractors, simulating different brightness perception with modulated responses.

Recent experimental observations have localized brightness perception to a site preceding binocular fusion^2^. Following the associated biological structure, our network can simulate both brightness perception and contextual modifications at the same time, providing a theoretical framework based on probabilistic inference to explore how distractors modulate neural responses and lead to different brightness perception of the same target. Our model provides an alternative method to explore brightness perception from contextual modification of primary visual cortical neural responses and interactions.

## Supplementary Information

Supplementary Information.

## Data Availability

Supporting codes and data will be made available upon request to the corresponding author.
