# Supplementary material for: The effects of distractors on brightness perception based on a spiking network
Source: Sci Rep. 2023 Jan 27;13:1517. doi: 10.1038/s41598-023-28326-4 (PMC9883501; doi:10.1038/s41598-023-28326-4)
Supplement: Supplementary file 1 — Supplementary Information. [file 41598_2023_28326_MOESM1_ESM.doc]

**Supplementary material**

**for:**

**The effects of distractors on brightness perception based on a spiking network**

Weisi Liu and Xinsheng Liu*

*Corresponding author. E-mail address: xsliu@nuaa.edu.cn.

State Key Laboratory of Mechanics and Control of Mechanical Structures, Institute of Nano Science and Department of Mathematics, Nanjing University of Aeronautics and Astronautics, Nanjing 210016, China

In this supplementary material, we provide the details of the network and the probabilistic model, as well as the identification of the stimuli. For convenience, the network structure is introduced in briefly.

***The structure of the spiking network***

Excitatory neurons in the second layer receive both feedforward and lateral inputs. There are $K$ second-layer excitatory neurons in the network. The temporal membrane potential of the $k$th second-layer excitatory neuron is given as^1^:

$u_{tk}^{z}=\sum_{n} w_{kn}\tilde{x}_{tn}+\sum_{k^{'}} v_{kk^{'}}\tilde{z}_{tk^{'}}-\sum_{j\in J_{k}} v^{EI}\tilde{y}_{tj},$ (S1)

where $u_{tk}^{z}$ is its membrane potential at timestep $t$, $w_{kn}\tilde{x}_{tn}$ is the instantaneous excitatory postsynaptic potentials (EPSP) coming from the $n$th afferent neuron. $w_{kn}$ is the corresponding plastic feedforward weight. Generated by corresponding second-layer excitatory neurons, $v_{kk^{'}}\tilde{z}_{tk^{'}}$ is the instantaneous lateral EPSP. $v_{kk^{'}}$ is the plastic lateral excitatory connective weight. All the inhibitory neurons connected to the $k$th excitatory neuron are collected into the set of $J_{k}$. Through random decisions of the neural inter-connections, the structure of the second-layer network accords with the observed basic neural circuit in the layer 2/3. The $v^{EI}\tilde{y}_{tj}$ is the instantaneous inhibitory postsynaptic potential (IPSP) coming from the $j$th inhibitory neuron among $J_{k}$. In the network, it is assumed that all the lateral weights from inhibitory neurons to excitatory ones are fixed, denoted as the common parameter $v^{EI}$.

The probabilistic distributions of $\tilde{z}_{tk'}$ and $\tilde{y}_{tj}$ could be expressed as the Dirac delta density distributions:

$\begin{matrix} p\left(\tilde{z}_{tk'}\left|\vec{\tilde{z}}_{t-1},\vec{z}_{t-1};\Theta\right.\right)=\delta\left(\tilde{z}_{tk'}-\sum_{t_{k'}^{(seq_{z})}\leq t} \varepsilon(t-t_{k'}^{(seq_{z})})\right) \\ p\left(\tilde{y}_{tj}\left|\vec{\tilde{y}}_{t-1},\vec{y}_{t-1};\Theta\right.\right)=\delta\left(\tilde{y}_{tj}-\sum_{t_{j}^{(seq_{y})}\leq t} \varepsilon(t-t_{j}^{(seq_{y})})\right) \end{matrix}$ (S2)

Whether a neuron generates a spike at the timestep $t$ or not is indicated by the temporal neural active state. The simulations in this paper are performed with a timestep of 1 millisecond. The timestep is small enough that each neuron is assumed to emit only one spike at most within each timestep. With the assumption, the temporal active state of the $k$th second-layer excitatory neuron is expressed as a variable $z_{tk}\in\left\{0,1\right\}$. The distribution of $z_{tk}$ depends on its temporal membrane potential as^1^:

$p\left(z_{tk}\left|\vec{\tilde{x}}_{t},\vec{\tilde{z}}_{t},\vec{\tilde{y}}_{t}\right.;\Theta\right)=\frac{\exp\left(u_{tk}^{z}z_{tk}\right)}{\sum_{z'_{tk}\in\left\{0,1\right\}} \exp\left(u_{tk}^{z}z'_{tk}\right)}=\frac{\exp\left[\left(\sum_{n} w_{kn}\tilde{x}_{tn}+\sum_{k^{'}} v_{kk^{'}}\tilde{z}_{tk^{'}}-\sum_{j\in J_{k}} v^{EI}\tilde{y}_{tj}\right)\cdot z_{tk}\right]}{\sum_{z'_{tk}\in\left\{0,1\right\}} \exp\left[\left(\sum_{n} w_{kn}\tilde{x}_{tn}+\sum_{k^{'}} v_{kk^{'}}\tilde{z}_{tk^{'}}-\sum_{j\in J_{k}} v^{EI}\tilde{y}_{tj}\right)\cdot z'_{tk}\right]},$ (S3)

where $u_{tk}^{z}$ is the membrane potential of the $k$th excitatory neuron, $z_{tk}$ and $z'_{tk}$ are the neural active states. In the denominator, $z'_{tk}$ takes all the possible values of the neural temporal active state for normalization. After generating a spike, the $k$th excitatory neuron can’t emit another spike again in a refractory period of 5 timesteps and $z_{tk}$ is set as 0.

The network could simulate identifications of stimuli at each timestep $t$ through the comparisons of the temporal second-layer excitatory neural responses $\vec{z}_{t}=\left(z_{t1},\cdots,z_{tK}\right)^{'}$ and the clustering sets.

The temporal membrane potential and the instantaneous firing rate of the $j$th inhibitory neuron can be expressed as ^2^:

(S4)

where $\tilde{z}_{tk}$ and $\tilde{y}_{tj'}$ are the temporal synaptic traces. $\rho_{tj}^{y}$ is the time-varying firing rate of the $j$th inhibitory neuron and $u_{tj}^{y}$ is the temporal membrane potential. With the given connective probabilities, the excitatory and inhibitory lateral connections towards the $j$th inhibitory neuron are determined stochastically. Indices of all the excitatory neurons in different neural population connecting to the $j$th inhibitory neuron are marked as . Indices of lateral inhibitory neurons connecting to the $j$th inhibitory neuron are collected as the set $\varsigma_{j}$. $v^{IE}\left(v^{II}\right)$ is the fixed weight from the lateral excitatory (inhibitory) neurons to the inhibitory ones. Through Poisson process, the $j$th inhibitory neuron generates its spikes with temporal responding strength of $\rho_{tj}^{y}$. Its absolute refractory period is of 3 timesteps. Its temporal active state is expressed as $y_{tj}\in\left\{0,1\right\}$. The distribution of $y_{tj}$ could be expressed as:

(S5)

***The probabilistic model***

The outside stimulus is marked as the variable $Sti$. From the beginning of the simulation, neurons in both two layers generate spiking responses to $Sti$. The temporal reward at timestep $t$ is $r_{t}$. Up to timestep $t$, the induced first-layer neural responses perform as the observed variables and are marked as the sequence of $\vec{O}_{t}=\left(\vec{o}_{1},\cdots,\vec{o}_{t}\right)$. The induced second-layer neural responses perform as the hidden variables and ae marked as $\vec{H}_{t}=\left(\vec{h}_{1},\cdots,\vec{h}_{t}\right)$. For the paired observed and hidden variables $\left\{\vec{O}_{t},\vec{H}_{t}\right\}$, as well as the outside stimulus $Sti$, a kind of Kullback-Leibler divergence is expressed as ^3^:

$\begin{matrix} D_{KL}\left\{p\left(\vec{O}_{t},\vec{H}_{t},Sti\left|r_{t}=1\right.\right)\left\|p\left(\vec{O}_{t},\vec{H}_{t},Sti;\Theta\right)\right.\right\} \\ = \iiint d\vec{O}_{t}d\vec{H}_{t}dSti\left[p\left(\vec{O}_{t},\vec{H}_{t},Sti\left|r_{t}=1\right.\right)\cdot\log\frac{p\left(\vec{O}_{t},\vec{H}_{t},Sti\left|r_{t}=1\right.\right)}{p\left(\vec{O}_{t},\vec{H}_{t},Sti;\Theta\right)}\right] \\ = -H_{p\left(\vec{O}_{t},\vec{H}_{t},Sti\left|r_{t}=1\right.\right)}-\left\langle\log p\left(\vec{O}_{t},\vec{H}_{t},Sti;\Theta\right)\right\rangle_{p\left(\vec{O}_{t},\vec{H}_{t},Sti\left|r_{t}=1\right.\right)}, \end{matrix}$ (S6)

where $r_{t}$ is the temporal reward at timestep $t$ and $\Theta$ is the set of parameters in the network. $r_{t}=1$ means the temporal identification of the stimulus is correct and the network receives the positive reward.

To minimize the Kullback-Leibler divergence $D_{KL}\left\{p\left(\vec{O}_{t},\vec{H}_{t},Sti\left|r_{t}=1\right.\right)\left\|p\left(\vec{O}_{t},\vec{H}_{t},Sti;\Theta\right)\right.\right\}$ is equal to maximize the term of $\left\langle\log p\left(\vec{O}_{t},\vec{H}_{t},Sti;\Theta\right)\right\rangle_{p\left(\vec{O}_{t},\vec{H}_{t},Sti\left|r_{t}=1\right.\right)}$. However, it is impossible to generate the neural activities before timestep $t$ from the unknown $r_{t}$ and the unknown distribution $p\left(\vec{O}_{t},\vec{H}_{t},Sti\left|r_{t}=1\right.\right)$. To overcome this problem, the maximization of $\left\langle\log p\left(\vec{O}_{t},\vec{H}_{t},Sti;\Theta\right)\right\rangle_{p\left(\vec{O}_{t},\vec{H}_{t},Sti\left|r_{t}=1\right.\right)}$ in this paper is implemented by maximizing another expectation as $\left\langle r_{t}\cdot\log p\left(\vec{O}_{t},\vec{H}_{t},Sti;\Theta\right)\right\rangle_{p\left(\vec{O}_{t},\vec{H}_{t},Sti,r_{t}\right)}$ because the relationship between them is:

$\begin{matrix} \left\langle r_{t}\cdot\log p\left(\vec{O}_{t},\vec{H}_{t},Sti;\Theta\right)\right\rangle_{p\left(\vec{O}_{t},\vec{H}_{t},Sti,r_{t}\right)} \\ = \iiint d\vec{O}_{t}d\vec{H}_{t}dSti\left[1\cdot\log p\left(\vec{O}_{t},\vec{H}_{t},Sti;\Theta\right)\cdot p\left(\vec{O}_{t},\vec{H}_{t},Sti,r_{t}=1\right)+0\cdot\log p\left(\vec{O}_{t},\vec{H}_{t},Sti;\Theta\right)\cdot p\left(\vec{O}_{t},\vec{H}_{t},Sti,r_{t}=0\right)\right] \\ = \iiint d\vec{O}_{t}d\vec{H}_{t}dSti\left[\log p\left(\vec{O}_{t},\vec{H}_{t},Sti;\Theta\right)\cdot p\left(\vec{O}_{t},\vec{H}_{t},Sti,r_{t}=1\right)\right] \\ \propto\iiint d\vec{O}_{t}d\vec{H}_{t}dSti\left[\log p\left(\vec{O}_{t},\vec{H}_{t},Sti;\Theta\right)\cdot p\left(\vec{O}_{t},\vec{H}_{t},Sti\left|r_{t}=1\right.\right)\right] \\ = \left\langle\log p\left(\vec{O}_{t},\vec{H}_{t},Sti;\Theta\right)\right\rangle_{p\left(\vec{O}_{t},\vec{H}_{t},Sti\left|r_{t}=1\right.\right)} \end{matrix}$ (S7)

It means that, for a sequence of dynamics from the beginning of the simulation to timestep $t$, the network modifies its parameters to maximize $\left\langle r_{t}\cdot\log p\left(\vec{O}_{t},\vec{H}_{t},Sti;\Theta\right)\right\rangle_{p\left(\vec{O}_{t},\vec{H}_{t},Sti,r_{t}\right)}$.

Up to timestep $t$, the paired observed and hidden variables $\left\{\vec{O}_{t},\vec{H}_{t}\right\}$ indicate all the neural responses over $t$ timesteps from the beginning of the simulation. The identification of the stimulus, as well at the reward, at $t$ is influenced by these neural responses. The lengths of $\vec{O}_{t}$ and $\vec{H}_{t}$ are considered to be $t$ and $\left\{\vec{O}_{t},\vec{H}_{t}\right\}$ are considered as $t$-step paired sequences. To consider the various influences of neural responding sequences with different lengths, the length-dependent rates are added. For $r_{t}=1$ at timestep t, there are several sub-sequences of dynamics contributing to the temporal identification, with different lengths expressed as $\tau\in\left\{1,\cdots,t\right\}$. With a given length of $\tau$, the observed variables and hidden variables up to time $t$ can be marked as $\vec{O}_{\tau}=\left(\vec{o}_{t-\tau+1},\cdots,\vec{o}_{t}\right),\vec{H}_{\tau}=\left(\vec{h}_{t-\tau+1},\cdots,\vec{h}_{t}\right)$. $\left\{\vec{O}_{t},\vec{H}_{t}\right\}$ are the paired sequences with the longest lengths. The contribute of the pair of $\tau$-step observed variables and hidden variables, $\left\{\vec{O}_{\tau},\vec{H}_{\tau}\right\}$, to $r_{t}$ is assumed as a rate $\alpha\left(\tau\right)=\left(1-\gamma\right)\gamma^{\tau-1}$ with the discount factor $\gamma\in\left(0,1\right)$. It should be noticed that $\left\{\alpha\left(\tau\right)>0,\tau=1,2,3,\cdots\right\}$ and $\sum_{\tau=1}^{\infty} \alpha\left(\tau\right)=1$. It follows the assumption that, the longer sequence of dynamics could be affected by previous information and make less contribution to the temporary reward $r_{t}=1$.

Based on the Kullback-Leibler divergence and the expectation, the likelihood function $L\left(\Theta\right)$ is designed with the contributions of all the neural responding sequences up to timestep $t$ as:

$L\left(\Theta\right)=\sum_{\tau=1}^{t} \left[\alpha\left(\tau\right)\cdot\left\langle r_{t}\cdot\log p\left(\vec{O}_{\tau},\vec{H}_{\tau},Sti;\Theta\right)\right\rangle_{p\left(\vec{O}_{\tau},\vec{H}_{\tau},Sti,r_{t}\right)}\right].$ (S8)

If only the paired $t$-step sequences $\left\{\vec{O}_{t},\vec{H}_{t}\right\}$ are considered, $L\left(\Theta\right)$ is reduced as $L\left(\Theta\right)=\alpha\left(t\right)\cdot\left\langle r_{t}\cdot\log p\left(\vec{O}_{t},\vec{H}_{t},Sti;\Theta\right)\right\rangle_{p\left(\vec{O}_{t},\vec{H}_{t},Sti,r_{t}\right)}$ with $\alpha\left(t\right)>0$. In this situation, the maximization of $L\left(\Theta\right)$ is equal to maximize the expectation $\left\langle r_{t}\cdot\log p\left(\vec{O}_{t},\vec{H}_{t},Sti;\Theta\right)\right\rangle_{p\left(\vec{O}_{t},\vec{H}_{t},Sti,r_{t}\right)}$ and minimize the Kullback-Leibler divergence $D_{KL}\left\{p\left(\vec{O}_{t},\vec{H}_{t},Sti\left|r_{t}=1\right.\right)\left\|p\left(\vec{O}_{t},\vec{H}_{t},Sti;\Theta\right)\right.\right\}$. In this paper, the likelihood function $L\left(\Theta\right)$ is designed with $\left\{\vec{O}_{\tau},\vec{H}_{\tau};\tau=1,\cdots,t\right\}$ considered. The network would modify its plastic parameters to minimize $L\left(\Theta\right)$.

The network generates its responses from the distribution $p\left(\vec{O}_{\tau},\vec{H}_{\tau},Sti,r_{t}\right)$ which could be expressed as:

$\begin{matrix} p\left(\vec{O}_{\tau},\vec{H}_{\tau},Sti,r_{t}\right)= p\left(r_{t}\left|\vec{O}_{\tau},\vec{H}_{\tau},Sti\right.\right)\cdot p\left(\vec{O}_{\tau},\vec{H}_{\tau},Sti_{t}\right) \\ = p\left(r_{t}\left|\vec{O}_{\tau},\vec{H}_{\tau},Sti\right.\right)\cdot p\left(\vec{H}_{\tau}\left|\vec{O}_{\tau},Sti_{t}\right.\right)\cdot p\left(\vec{O}_{\tau},Sti_{t}\right) \\ = p\left(r_{t}\left|\vec{O}_{\tau},\vec{H}_{\tau},Sti\right.\right)\cdot p\left(\vec{H}_{\tau}\left|\vec{O}_{\tau},Sti_{t}\right.\right)\cdot p\left(\vec{O}_{\tau}\left|Sti_{t}\right.\right)\cdot p\left(Sti_{t}\right) \end{matrix}$ (S9)

Due to the relationship and the conditional independence between variables, $p\left(\vec{O}_{\tau},\vec{H}_{\tau},Sti,r_{t}\right)$ is simplified as:

$p\left(\vec{O}_{\tau},\vec{H}_{\tau},Sti,r_{t}\right)= p\left(r_{t}\left|\vec{H}_{\tau}\right.\right)\cdot p\left(\vec{H}_{\tau}\left|\vec{O}_{\tau}\right.\right)\cdot p\left(\vec{O}_{\tau}\left|Sti\right.\right)\cdot p\left(Sti\right)$ (S10)

The stimuli in our simulations are designed and $p\left(Sti\right)$ is the discrete distribution of equal probability. The first-layer neural synaptic traces are generated from $p\left(\vec{O}_{\tau}\left|Sti\right.\right)$. Each afferent neuron in the first layer generates its Poisson spikes with its received stimuli, independently. The network would receive the reward according to the identifications of stimuli with its second-layer neural activities. It indicates that $p\left(r_{t}\left|\vec{H}_{\tau}\right.\right)$ depends on the network responses and clustering sets.

The neural responses in the second-layer network are sampled from $p\left(\vec{H}_{\tau}\left|\vec{O}_{\tau}\right.\right)$. Due to the designed network structure, the second-layer neural responses at each timestep depend on the current feedforward inputs and lateral inputs from second-layer neural responses at previous timestep. Considering this dependence, $p\left(\vec{H}_{\tau}\left|\vec{O}_{\tau}\right.\right)$ is written as:

$\begin{matrix} p\left(\vec{H}_{\tau}\left|\vec{O}_{\tau}\right.\right)= \prod_{t'=t-\tau+1}^{t} p\left(\vec{h}_{t'}\left|\vec{o}_{t'},\vec{h}_{t'-1}\right.\right) \\ = \prod_{t'=t-\tau+1}^{t} \frac{p\left(\vec{o}_{t'},\vec{h}_{t'},\vec{h}_{t'-1}\right)}{p\left(\vec{o}_{t'},\vec{h}_{t'-1}\right)} \\ = \prod_{t'=t-\tau+1}^{t} \frac{p\left(\vec{o}_{t'}\left|\vec{h}_{t'},\vec{h}_{t'-1}\right.\right)p\left(\vec{h}_{t'},\vec{h}_{t'-1}\right)}{p\left(\vec{o}_{t'},\vec{h}_{t'-1}\right)} \end{matrix}$ (S11)

where $p\left(\vec{H}_{\tau}\left|\vec{O}_{\tau}\right.\right)$ describes the distributions of second-layer neural responses induced by the afferent neural activities. With the assumption of conditional independence, $p\left(\vec{o}_{t'}\left|\vec{h}_{t'},\vec{h}_{t'-1}\right.\right)\simeq p\left(\vec{o}_{t'}\left|\vec{h}_{t'}\right.\right)$ and $p\left(\vec{H}_{\tau}\left|\vec{O}_{\tau}\right.\right)$ is approximated as:

$\begin{matrix} p\left(\vec{H}_{\tau}\left|\vec{O}_{\tau}\right.\right)\simeq\prod_{t'=t-\tau+1}^{t} \frac{p\left(\vec{o}_{t'}\left|\vec{h}_{t'}\right.\right)p\left(\vec{h}_{t'},\vec{h}_{t'-1}\right)}{p\left(\vec{o}_{t'},\vec{h}_{t'-1}\right)} \\ = \prod_{t'=t-\tau+1}^{t} \frac{p\left(\vec{o}_{t'}\left|\vec{h}_{t'}\right.\right)p\left(\vec{h}_{t'}\left|\vec{h}_{t'-1}\right.\right)}{p\left(\vec{o}_{t'}\left|\vec{h}_{t'-1}\right.\right)} \end{matrix}$ (S12)

On the other hand, under the assumption of the hidden Markov model, $p\left(\vec{H}_{\tau}\left|\vec{O}_{\tau}\right.\right)$ could be factorized as:

$\begin{matrix} p\left(\vec{H}_{\tau}\left|\vec{O}_{\tau}\right.\right)= \frac{p\left(\vec{O}_{\tau},\vec{H}_{\tau}\right)}{p\left(\vec{O}_{\tau}\right)} \\ = \frac{p\left(\vec{O}_{\tau},\vec{H}_{\tau}\right)}{\int p\left(\vec{O}_{\tau},\vec{H}_{\tau}\right)d\vec{H}_{\tau}} \\ = \frac{\prod_{t'=t-t+1}^{t} p\left(\vec{o}_{t'}\left|\vec{h}_{t'}\right.\right)p\left(\vec{h}_{t'}\left|\vec{h}_{t'-1}\right.\right)}{\int\prod_{t'=t-t+1}^{t} p\left(\vec{o}_{t'}\left|\vec{h}_{t'}\right.\right)p\left(\vec{h}_{t'}\left|\vec{h}_{t'-1}\right.\right)d\vec{H}_{\tau}} \\ = \frac{\prod_{t'=t-t+1}^{t} p\left(\vec{o}_{t'}\left|\vec{h}_{t'}\right.\right)p\left(\vec{h}_{t'}\left|\vec{h}_{t'-1}\right.\right)}{\prod_{t'=t-t+1}^{t} \left[\int p\left(\vec{o}_{t'}\left|\vec{h}_{t'}\right.\right)p\left(\vec{h}_{t'}\left|\vec{h}_{t'-1}\right.\right)dt'\right]} \\ = \prod_{t'=t-t+1}^{t} \frac{p\left(\vec{o}_{t'}\left|\vec{h}_{t'}\right.\right)p\left(\vec{h}_{t'}\left|\vec{h}_{t'-1}\right.\right)}{p\left(\vec{o}_{t'}\left|\vec{h}_{t'-1}\right.\right)} \end{matrix}$ (S13)

Comparing two expressions, it indicates that the designed network could approximate the forward sampling in a Hidden Markov model:

$\begin{matrix} p\left(\vec{H}_{\tau}\left|\vec{O}_{\tau}\right.\right)= \prod_{t'=t-\tau+1}^{t} p\left(\vec{h}_{t'}\left|\vec{o}_{t'},\vec{h}_{t'-1}\right.\right) \\ \simeq\prod_{t'=t-t+1}^{t} \frac{p\left(\vec{o}_{t'}\left|\vec{h}_{t'}\right.\right)p\left(\vec{h}_{t'}\left|\vec{h}_{t'-1}\right.\right)}{p\left(\vec{o}_{t'}\left|\vec{h}_{t'-1}\right.\right)} \end{matrix}$ (S14)

Neural responses at each timestep $t'$ in the paired sub-sequences $\left\{\vec{O}_{\tau},\vec{H}_{\tau}\right\}$ are sampled from $p\left(\vec{h}_{t'}\left|\vec{o}_{t'},\vec{h}_{t'-1}\right.\right)$:

$\begin{matrix} p\left(\vec{h}_{t'}\left|\vec{o}_{t'},\vec{h}_{t'-1}\right.\right)= p\left(\vec{z}_{t'},\vec{\tilde{z}}_{t'},\vec{y}_{t'},\vec{\tilde{y}}_{t'}\left|\vec{\tilde{x}}_{t'},\vec{z}_{t'-1},\vec{\tilde{z}}_{t'-1},\vec{y}_{t'-1},\vec{\tilde{y}}_{t'-1}\right.\right) \\ = p\left(\vec{z}_{t'}\left|\vec{\tilde{x}}_{t'},\vec{\tilde{z}}_{t'},\vec{\tilde{y}}_{t'}\right.\right)\cdot p\left(\vec{y}_{t'}\left|\vec{\tilde{z}}_{t'},\vec{\tilde{y}}_{t'}\right.\right)\cdot p\left(\vec{\tilde{z}}_{t'}\left|\vec{z}_{t'-1},\vec{\tilde{z}}_{t'-1}\right.\right)\cdot p\left(\vec{\tilde{y}}_{t'}\left|\vec{y}_{t'-1},\vec{\tilde{y}}_{t'-1}\right.\right) \end{matrix}$ (S15)

In this paper, the second-layer neurons communicate each other through lateral inputs which could influence neural membrane potentials and neural firing rates. With neural firing rates given, neurons are assumed to generate spikes, independently. The neural synaptic traces are determined by the synaptic kernel function $\varepsilon$ and independent with each other. With these assumptions, $p\left(\vec{z}_{t'}\left|\vec{\tilde{x}}_{t'},\vec{\tilde{z}}_{t'},\vec{\tilde{y}}_{t'}\right.\right)=\prod_{k} p\left(z_{t'k}\left|\vec{\tilde{x}}_{t'},\vec{\tilde{z}}_{t'},\vec{\tilde{y}}_{t'}\right.\right)$, $p\left(\vec{y}_{t'}\left|\vec{\tilde{z}}_{t'},\vec{\tilde{y}}_{t'}\right.\right)=\prod_{j} p\left(y_{t'j}\left|\vec{\tilde{z}}_{t'},\vec{\tilde{y}}_{t'}\right.\right)$, $p\left(\vec{\tilde{z}}_{t'}\left|\vec{z}_{t'-1},\vec{\tilde{z}}_{t'-1}\right.\right)=\prod_{k'} p\left(\tilde{z}_{tk'}\left|\vec{\tilde{z}}_{t-1},\vec{z}_{t-1}\right.\right)$, $p\left(\vec{\tilde{y}}_{t'}\left|\vec{y}_{t'-1},\vec{\tilde{y}}_{t'-1}\right.\right)=\prod_{j} p\left(\tilde{y}_{tj}\left|\vec{\tilde{y}}_{t-1},\vec{y}_{t-1}\right.\right)$.

With the distributions expressed above, the neural responses sampled from $p\left(\vec{O}_{\tau},\vec{H}_{\tau},Sti,r_{t}\right)$ could be used to calculate the likelihood function $L\left(\Theta\right)$. Under the assumption of Hidden Markov model, the joint distributions $p\left(\vec{O}_{\tau},\vec{H}_{\tau},Sti;\Theta\right)$ in $L\left(\Theta\right)$ could be factorized as:

$\begin{matrix} p\left(\vec{O}_{\tau},\vec{H}_{\tau},Sti;\Theta\right) \\ = p\left(Sti\left|\vec{O}_{\tau},\vec{H}_{\tau};\Theta\right.\right)p\left(\vec{O}_{\tau},\vec{H}_{\tau};\Theta\right) \\ = p\left(Sti\left|\vec{O}_{\tau},\vec{H}_{\tau};\Theta\right.\right)\cdot\prod_{t'=t-\tau+1}^{t} p\left(\vec{o}_{t'}\left|\vec{h}_{t'};\Theta\right.\right)p\left(\vec{h}_{t'}\left|\vec{h}_{t'-1};\Theta\right.\right). \end{matrix}$ (S16)

With the factorization, $L\left(\Theta\right)$ could be expressed as:

$\begin{matrix} L\left(\Theta\right)= \sum_{\tau=1}^{t} \left\{\alpha\left(\tau\right)\cdot\left\langle r_{t}\cdot\log\left[p\left(Sti\left|\vec{O}_{\tau},\vec{H}_{\tau};\Theta\right.\right)\cdot\prod_{t'=t-\tau+1}^{t} p\left(\vec{o}_{t'}\left|\vec{h}_{t'};\Theta\right.\right)p\left(\vec{h}_{t'}\left|\vec{h}_{t'-1};\Theta\right.\right)\right]\right\rangle_{p\left(\vec{O}_{\tau},\vec{H}_{\tau},Sti,r_{t}\right)}\right\} \\ = \sum_{\tau=1}^{t} \alpha\left(\tau\right)\cdot\left\langle r_{t}\cdot\log p\left(Sti\left|\vec{O}_{\tau},\vec{H}_{\tau};\Theta\right.\right)+r_{t}\cdot\sum_{t'=t-\tau+1}^{t} \log\left[p\left(\vec{o}_{t'}\left|\vec{h}_{t'};\Theta\right.\right)p\left(\vec{h}_{t'}\left|\vec{h}_{t'-1};\Theta\right.\right)\right]\right\rangle_{p\left(\vec{O}_{\tau},\vec{H}_{\tau},Sti,r_{t}\right)}. \end{matrix}$(S17)

The network optimizes its parameters through the stochastic online variant of the Expectation-Maximization algorithm, in which the expectation is estimated during the E-step by sampling a finite set of samples. For each pair of sub-sequences $\left\{\vec{O}_{\tau},\vec{H}_{\tau}\right\}$ in our simulations, the E-step is to estimate the expectation with a single sample from $p\left(\vec{O}_{\tau},\vec{H}_{\tau},Sti,r_{t}\right)$. Then, the likelihood function $L\left(\Theta\right)$ is approximated as:

$L\left(\Theta\right)\simeq\sum_{\tau=1}^{t} \alpha\left(\tau\right)\cdot r_{t}\cdot\left[\log p\left(Sti\left|\vec{O}_{\tau},\vec{H}_{\tau};\Theta\right.\right)+\sum_{t'=t-\tau+1}^{t} \log p\left(\vec{o}_{t'}\left|\vec{h}_{t'};\Theta\right.\right)p\left(\vec{h}_{t'}\left|\vec{h}_{t'-1};\Theta\right.\right)\right].$(S18)

The terms of $\left\{\log p\left(Sti\left|\vec{O}_{\tau},\vec{H}_{\tau};\Theta\right.\right),\tau=1,\cdots,t\right\}$ indicate the identifications of the outside stimuli with the induced network responses. In this paper, the identifications depend on the clustering sets and $\left\{\log p\left(Sti\left|\vec{O}_{\tau},\vec{H}_{\tau};\Theta\right.\right),\tau=1,\cdots,t\right\}$ are independent of the connective weights. The term of $\sum_{t'=t-\tau+1}^{t} \log p\left(\vec{o}_{t'}\left|\vec{h}_{t'};\Theta\right.\right)p\left(\vec{h}_{t'}\left|\vec{h}_{t'-1};\Theta\right.\right)$ is associated with plastic connective weights. For a given $t'$, the term $\log p\left(\vec{o}_{t'}\left|\vec{h}_{t'};\Theta\right.\right)p\left(\vec{h}_{t'}\left|\vec{h}_{t'-1};\Theta\right.\right)$ multiplies different rates $\alpha$. The likelihood function $L\left(\Theta\right)$ is rearranged and approximated as:

$\begin{matrix} L\left(\Theta\right)= r_{t}\cdot\sum_{t'=1}^{t} \log\left[p\left(\vec{o}_{t'}\left|\vec{h}_{t'};\Theta\right.\right)p\left(\vec{h}_{t'}\left|\vec{h}_{t'-1};\Theta\right.\right)\right]\cdot\left[\sum_{\tau=t-t'+1}^{t} \alpha\left(\tau\right)\right]+r_{t}\cdot\sum_{\tau=1}^{t} \alpha\left(\tau\right)\log p\left(Sti\left|\vec{O}_{\tau},\vec{H}_{\tau};\Theta\right.\right) \\ \simeq r_{t}\cdot\sum_{t'=1}^{t} \log\left[p\left(\vec{o}_{t'}\left|\vec{h}_{t'};\Theta\right.\right)p\left(\vec{h}_{t'}\left|\vec{h}_{t'-1};\Theta\right.\right)\right]\cdot\left[\sum_{\tau=t-t'+1}^{\infty} \alpha\left(\tau\right)\right]+r_{t}\cdot\sum_{\tau=1}^{t} \alpha\left(\tau\right)\log p\left(Sti\left|\vec{O}_{\tau},\vec{H}_{\tau};\Theta\right.\right) \\ = r_{t}\cdot\sum_{t'=1}^{t} \log\left[p\left(\vec{o}_{t'}\left|\vec{h}_{t'};\Theta\right.\right)p\left(\vec{h}_{t'}\left|\vec{h}_{t'-1};\Theta\right.\right)\right]\cdot\left[1-\sum_{\tau=1}^{t-t'} \alpha\left(\tau\right)\right]+r_{t}\cdot\sum_{\tau=1}^{t} \alpha\left(\tau\right)\log p\left(Sti\left|\vec{O}_{\tau},\vec{H}_{\tau};\Theta\right.\right) \\ = r_{t}\cdot\sum_{t'=1}^{t} \gamma^{t-t'}\cdot\left[\log p\left(\vec{o}_{t'}\left|\vec{h}_{t'};\Theta\right.\right)+\log p\left(\vec{h}_{t'}\left|\vec{h}_{t'-1};\Theta\right.\right)\right]+r_{t}\cdot\sum_{\tau=1}^{t} \alpha\left(\tau\right)\log p\left(Sti\left|\vec{O}_{\tau},\vec{H}_{\tau};\Theta\right.\right). \end{matrix}$(S19)

The observation model is:

$p\left(\vec{o}_{t'}\left|\vec{h}_{t'};\Theta\right.\right)= p\left(\vec{\tilde{x}}_{t'}\left|\vec{z}_{t'},\vec{\tilde{z}}_{t'},\vec{y}_{t'},\vec{\tilde{y}}_{t'};\Theta\right.\right)= p\left(\vec{\tilde{x}}_{t'}\left|\vec{z}_{t'};\Theta\right.\right)$ (S20)

The afferent neurons in the first layer are assumed to simulate retinal neural responses, independently. The joint distribution $p\left(\vec{\tilde{x}}_{t'}\left|\vec{z}_{t'};\Theta\right.\right)$ is factorized as:

$\begin{matrix} p\left(\vec{\tilde{x}}_{t'}\left|\vec{z}_{t'};\Theta\right.\right) =\prod_{n} p\left(\tilde{x}_{t'n}\left|\vec{z}_{t'};\Theta\right.\right) \\ p\left(\tilde{x}_{t'n}\left|\vec{z}_{t'};\Theta\right.\right) =\frac{p\left(\tilde{x}_{t'n},\vec{z}_{t'};\Theta\right)}{p\left(\vec{z}_{t'};\Theta\right)}=\frac{p\left(\tilde{x}_{t'n},\vec{z}_{t'};\Theta\right)}{\int p\left(\tilde{x}_{t'n},\vec{z}_{t'};\Theta\right)d\tilde{x}_{t'n}} \\ p\left(\tilde{x}_{t'n},\vec{z}_{t'};\Theta\right) =\frac{1}{C}\exp\left(\sum_{k} w_{kn}\tilde{x}_{t'n}z_{t'k}\right) \\ C =\sum_{\left\{z'_{t'k}\in\left\{0,1\right\},k=1,\cdots,K\right\}} \left[\int\exp\left(\sum_{k} w_{kn}\tilde{x}'_{t'n}z'_{t'k}\right)d\tilde{x}'_{t'n}\right] \end{matrix}$ (S21)

where $z'_{tk}$ and $\tilde{x}'_{tn}$ in the term $C$ take all the possible values for normalization. Then, $p\left(\vec{\tilde{x}}_{t'}\left|\vec{z}_{t'};\Theta\right.\right)$ could expressed as:

$p\left(\tilde{x}_{t'n}\left|\vec{z}_{t'};\Theta\right.\right)= \frac{\exp\left(\sum_{k} w_{kn}\tilde{x}_{t'n}z_{t'k}\right)}{\int\exp\left(\sum_{k} w_{kn}\tilde{x}_{t'n}z_{t'k}\right)d\tilde{x}_{t'n}}$ (S22)

The predictive model is:

$\begin{matrix} p\left(\vec{h}_{t'}\left|\vec{h}_{t'-1};\Theta\right.\right)= p\left(\vec{z}_{t'},\vec{\tilde{z}}_{t'},\vec{y}_{t'},\vec{\tilde{y}}_{t'}\left|\vec{z}_{t'-1},\vec{\tilde{z}}_{t'-1},\vec{y}_{t'-1},\vec{\tilde{y}}_{t'-1};\Theta\right.\right) \\ = p\left(\vec{z}_{t'}\left|\vec{\tilde{z}}_{t'},\vec{\tilde{y}}_{t'};\Theta\right.\right)\cdot p\left(\vec{y}_{t'}\left|\vec{\tilde{z}}_{t'},\vec{\tilde{y}}_{t'};\Theta\right.\right)\cdot p\left(\vec{\tilde{z}}_{t'}\left|\vec{z}_{t'-1},\vec{\tilde{z}}_{t'-1}\right.\right)\cdot p\left(\vec{\tilde{y}}_{t'}\left|\vec{y}_{t'-1},\vec{\tilde{y}}_{t'-1}\right.\right) \end{matrix}$(S23)

where $p\left(\vec{\tilde{z}}_{t'}\left|\vec{z}_{t'-1},\vec{\tilde{z}}_{t'-1}\right.\right)$ and $p\left(\vec{\tilde{y}}_{t'}\left|\vec{y}_{t'-1},\vec{\tilde{y}}_{t'-1}\right.\right)$ are the Dirac delta density distributions which are independent of plastic parameters. $p\left(\vec{y}_{t'}\left|\vec{\tilde{z}}_{t'},\vec{\tilde{y}}_{t'};\Theta\right.\right)=\prod_{j} p\left(y_{t'j}\left|\vec{\tilde{z}}_{t'},\vec{\tilde{y}}_{t'};\Theta\right.\right)$ is independent of plastic parameters.

$\begin{matrix} p\left(\vec{z}_{t'}\left|\vec{\tilde{z}}_{t'},\vec{\tilde{y}}_{t'};\Theta\right.\right)=\frac{p\left(\vec{z}_{t'},\vec{\tilde{z}}_{t'},\vec{\tilde{y}}_{t'};\Theta\right)}{p\left(\vec{\tilde{z}}_{t'},\vec{\tilde{y}}_{t'};\Theta\right)} \\ p\left(\vec{z}_{t'},\vec{\tilde{z}}_{t'},\vec{\tilde{y}}_{t'};\Theta\right)=\frac{\exp\left[\sum_{k} z_{tk}\cdot\left(\sum_{k'} v_{kk'}\tilde{z}_{tk'}-\sum_{j'\in J_{k}} v^{EI}\tilde{y}_{tj'}\right)\right]}{\sum_{\left\{z'_{tk}\in\left\{0,1\right\},k=1,\cdots,K\right\}} \iint\exp\left[\sum_{k} z_{tk}\cdot\left(\sum_{k'} v_{kk'}\tilde{z}_{tk'}-\sum_{j'\in J_{k}} v^{EI}\tilde{y}_{tj'}\right)\right]d\vec{\tilde{z}}'_{t}d\vec{\tilde{y}}'_{t}} \end{matrix}$ (S24)

$p\left(\vec{\tilde{z}}_{t'},\vec{\tilde{y}}_{t'};\Theta\right)$ is independent of plastic parameters.

Along the directions given by the partial derivatives of $L\left(\Theta\right)$, the network optimizes the plastic connective weights in the M-step. Specifically, with respect to a plastic feedforward weight $w_{kn}$, the direction of modification is given by:

$\begin{matrix} \frac{\partial}{\partial w_{kn}}L\left(\Theta\right) \\ = \frac{\partial}{\partial w_{kn}}r_{t}\cdot\sum_{t'=1}^{t} \gamma^{t-t'}\cdot\log p\left(\vec{o}_{t'}\left|\vec{h}_{t'};\Theta\right.\right) \\ = r_{t}\sum_{t'=1}^{t} \gamma^{t-t'}\frac{\partial}{\partial w_{kn}}\log p\left(\tilde{x}_{t'n}\left|\vec{z}_{t'};\Theta\right.\right) \\ = r_{t}\sum_{t'=1}^{t} \gamma^{t-t'}\frac{\partial}{\partial w_{kn}}\left\{\left(\sum_{k} w_{kn}\tilde{x}_{t'n}z_{t'k}\right)-\log\left[\int\exp\left(\sum_{k} w_{kn}\tilde{x}_{t'n}z_{t'k}\right)d\tilde{x}_{t'n}\right]\right\} \\ = r_{t}\sum_{t'=1}^{t} \gamma^{t-t'}\left\{\tilde{x}_{t'n}z_{t'k}-\frac{\partial}{\partial w_{kn}}\log\left[\int\exp\left(\sum_{k} w_{kn}\tilde{x}_{t'n}z_{t'k}\right)d\tilde{x}_{t'n}\right]\right\} \end{matrix}$ (S25)

Particularly, $\frac{\partial}{\partial w_{kn}}\log\left[\int\exp\left(\sum_{k} w_{kn}\tilde{x}_{t'n}z_{t'k}\right)d\tilde{x}_{t'n}\right]$ could be expressed as:

$\begin{matrix} \frac{\partial}{\partial w_{kn}}\log\left[\int\exp\left(\sum_{k} w_{kn}\tilde{x}_{t'n}z_{t'k}\right)d\tilde{x}_{t'n}\right] \\ = \frac{\frac{\partial}{\partial w_{kn}}\int\exp\left(\sum_{k} w_{kn}\tilde{x}_{t'n}z_{t'k}\right)d\tilde{x}_{t'n}}{\int\exp\left(\sum_{k} w_{kn}\tilde{x}_{t'n}z_{t'k}\right)d\tilde{x}_{t'n}} \\ = \frac{\frac{\partial}{\partial w_{kn}}\int\prod_{k} \exp\left(w_{kn}\tilde{x}_{t'n}z_{t'k}\right)d\tilde{x}_{t'n}}{\int\prod_{k} \exp\left(w_{kn}\tilde{x}_{t'n}z_{t'k}\right)d\tilde{x}_{t'n}} \\ = \frac{\int\left[\frac{\partial}{\partial w_{kn}}\prod_{k} \exp\left(w_{kn}\tilde{x}_{t'n}z_{t'k}\right)\right]d\tilde{x}_{t'n}}{\int\prod_{k} \exp\left(w_{kn}\tilde{x}_{t'n}z_{t'k}\right)d\tilde{x}_{t'n}} \end{matrix}$ (S26)

To simplify the expression, the partial derivative in the numerator is re-arranged and re-expressed as:

$\begin{matrix} \frac{\partial}{\partial w_{kn}}\prod_{k} \exp\left(w_{kn}\tilde{x}_{t'n}z_{t'k}\right) \\ = \prod_{k'\neq k} \exp\left(w_{k'n}\tilde{x}_{t'n}z_{t'k'}\right)\cdot\frac{\partial}{\partial w_{kn}}\exp\left(w_{kn}\tilde{x}_{t'n}z_{t'k}\right) \\ = \prod_{k'\neq k} \exp\left(w_{k'n}\tilde{x}_{t'n}z_{t'k'}\right)\cdot\exp\left(w_{kn}\tilde{x}_{t'n}z_{t'k}\right)\cdot\tilde{x}_{t'n}\cdot z_{t'k} \\ = z_{t'k}\cdot\exp\left(\sum_{k} w_{kn}\tilde{x}_{t'n}z_{t'k}\right)\cdot\tilde{x}_{t'n} \end{matrix}$ (S27)

Then, $\frac{\partial}{\partial w_{kn}}\log\left[\int\exp\left(\sum_{k} w_{kn}\tilde{x}_{t'n}z_{t'k}\right)d\tilde{x}_{t'n}\right]$ could be re-expressed as:

$\frac{\partial}{\partial w_{kn}}\log\left[\int\exp\left(\sum_{k} w_{kn}\tilde{x}_{t'n}z_{t'k}\right)d\tilde{x}_{t'n}\right]= \frac{z_{t'k}\cdot\int\left[\exp\left(\sum_{k} w_{kn}\tilde{x}_{t'n}z_{t'k}\right)\cdot\tilde{x}_{t'n}\right]d\tilde{x}_{t'n}}{\int\exp\left(\sum_{k} w_{kn}\tilde{x}_{t'n}z_{t'k}\right)d\tilde{x}_{t'n}}$ (S28)

It should be noticed that each $\tilde{x}_{t'n}$ in our simulations is limitary. $\left\{\tilde{x}_{t'n},n=1,\cdots,N\right\}$ are assumed to have the common maximum $\tilde{x}_{max}$ and the common minimum $\tilde{x}_{min}$. The term $\sum_{k} w_{kn}z_{t'k}$ is marked as $\beta_{t'n}$ and $\exp\left(\sum_{k} w_{kn}\tilde{x}_{t'n}z_{t'k}\right)$ is marked as $\exp\left(\beta_{t'n}\tilde{x}_{t'n}\right)$. The integrals should be definite and are calculated as:

$\begin{matrix} \int_{\tilde{x}_{min}}^{\tilde{x}_{max}} \exp\left(\beta_{t'n}\tilde{x}_{t'n}\right)\cdot\tilde{x}_{t'n}d\tilde{x}_{t'n} \\ = \int_{\tilde{x}_{min}}^{\tilde{x}_{max}} \tilde{x}_{t'n}d\left[\exp\left(\beta_{t'n}\tilde{x}_{t'n}\right)\right]/\beta_{t'n} \\ = \left\{\left[\exp\left(\beta_{t'n}\tilde{x}_{t'n}\right)\tilde{x}_{t'n}\right]_{\tilde{x}_{min}}^{\tilde{x}_{max}}-\int_{\tilde{x}_{min}}^{\tilde{x}_{max}} \exp\left(\beta_{t'n}\tilde{x}_{t'n}\right)d\tilde{x}_{t'n}\right\}/\beta_{t'n} \\ = 1/\beta_{t'n}\cdot\left[\exp\left(\beta_{t'n}\tilde{x}_{max}\right)\tilde{x}_{max}-\exp\left(\beta_{t'n}\tilde{x}_{min}\right)\tilde{x}_{min}\right]-1/\beta_{t'n}^{2}\cdot\left[\exp\left(\beta_{t'n}\tilde{x}_{max}\right)-\exp\left(\beta_{t'n}\tilde{x}_{min}\right)\right] \\ \int_{\tilde{x}_{min}}^{\tilde{x}_{max}} \exp\left(\beta_{t'n}\tilde{x}_{t'n}\right)d\tilde{x}_{t'n}=1/\beta_{t'n}\cdot\left[\exp\left(\beta_{t'n}\tilde{x}_{max}\right)-\exp\left(\beta_{t'n}\tilde{x}_{min}\right)\right] \end{matrix}$(S29)

In our simulations, $\tilde{x}_{max}=1$ and $\tilde{x}_{min}=0$. With the definite integrals, $\frac{\partial}{\partial w_{kn}}\log\left[\int_{\tilde{x}_{min}}^{\tilde{x}_{max}} \exp\left(\sum_{k} w_{kn}\tilde{x}_{t'n}z_{t'k}\right)d\tilde{x}_{t'n}\right]$ could be calculated as:

$\begin{matrix} \frac{\partial}{\partial w_{kn}}\log\left[\int_{\tilde{x}_{min}}^{\tilde{x}_{max}} \exp\left(\sum_{k} w_{kn}\tilde{x}_{t'n}z_{t'k}\right)d\tilde{x}_{t'n}\right] \\ = z_{t'k}\cdot\left\{\tilde{x}_{max}-\frac{1}{\sum_{k} w_{kn}\tilde{x}_{t'n}z_{t'k}}+\left(\tilde{x}_{max}-\tilde{x}_{min}\right)\cdot\frac{1}{\exp\left[\left(\tilde{x}_{max}-\tilde{x}_{min}\right)\cdot\left(\sum_{k} w_{kn}\tilde{x}_{t'n}z_{t'k}\right)\right]-1}\right\} \end{matrix}$(S30)

In order to make these learning rules more biologically plausible, we make the approximations similarly to a previous study ^4^. It is assumed that the modification of each weight only depends on its current value and independent with other weights. With this assumption, the partial derivatives of $L\left(\Theta\right)$ respect to $w_{kn}$ is expressed as:

$\begin{matrix} \frac{\partial}{\partial w_{kn}}L\left(\Theta\right) \\ = r_{t}\sum_{t'=1}^{t} \gamma^{t-t'}z_{t'k}\cdot\left\{\tilde{x}_{t'n}-\left[1-\frac{1}{\sum_{k} w_{kn}z_{t'k}}+\frac{1}{\exp\left(\sum_{k} w_{kn}z_{t'k}\right)-1}\right]\right\} \end{matrix}$ (S31)

The modification of $w_{kn}$ is expressed as:

$\begin{matrix} \Delta w_{kn}= \eta\cdot\frac{\partial}{\partial w_{kn}}L\left(\Theta\right) \\ \simeq\eta\cdot r_{t}\sum_{t'=1}^{t} \gamma^{t-t'}\cdot z_{t'k}\cdot\left\{\tilde{x}_{t'n}-\left[1-\frac{1}{w_{kn}}+\frac{1}{\exp\left(w_{kn}\right)-1}\right]\right\} \end{matrix}$ (S32)

where $\eta$ is the learning rate. In our simulations, $\eta=0.005$.

Similarly, the modification of a plastic lateral weight $v_{kk'}$ is:

$\begin{matrix} \Delta v_{kk'}= \eta\cdot\frac{\partial}{\partial v_{kk'}}L\left(\Theta\right) \\ \simeq\eta\cdot r_{t}\sum_{t'=1}^{t} \gamma^{t-t'}\cdot z_{t'k}\cdot\left\{\tilde{z}_{t'k'}-\left[1-\frac{1}{v_{kk'}}+\frac{1}{\exp\left(v_{kk'}\right)-1}\right]\right\} \end{matrix}$ (S33)

***Unsupervised identifications of outside stimuli***

This paper identifies the stimuli with second-layer excitatory spikes through an unsupervised online method ^5^. This method has no constraint conditions for the dimension of neural spikes and is suitable for the network with different sizes. Using this method, the network could identify the stimuli at each time step and receive the reward to control connective modifications.

The unsupervised identification depends on the distance between two sets of vectors. Generally speaking, for any two sets of vectors $A=\left(\vec{a}_{1},\cdots,\vec{a}_{n_{A}}\right)$ and $B=\left(\vec{b}_{1},\cdots,\vec{b}_{n_{B}}\right)$, the energy distance $e\left(A,B\right)$ between them can be calculated as:

$e\left(A,B\right) =\frac{n_{A}n_{B}}{n_{A}+n_{B}}(\frac{2}{n_{A}n_{B}}\sum_{i1=1}^{n_{A}} \sum_{j1=1}^{n_{B}} \left\|a_{i1}-b_{j1}\right\|-\frac{1}{n_{A}^{2}}\sum_{i2=1}^{n_{A}} \sum_{j2=1}^{n_{A}} \left\|a_{i2}-a_{j2}\right\|-\frac{1}{n_{B}^{2}}\sum_{i3=1}^{n_{B}} \sum_{j3=1}^{n_{B}} \left\|b_{i3}-b_{j3}\right\|)$ (S34)

where $n_{A},n_{B}$ are the sizes of $A$ and $B$，$a_{i1},b_{j1},a_{i2},a_{j2},b_{i3},b_{j3}$ indicate the elements in two sets. With the energy distance, the likelihood of the distributions of two sets being equal could be estimated.

For a set $B$ and several sets $\left\{A_{s},s=1,\cdots,S\right\}$, the identification of $B$ includes two steps based on the energy distance. In the first step, the likelihood between $B$ and each $A_{s}$ could be calculated. Based on these likelihoods, $B$ is identified as belonging to a certain cluster in the second step.

In the first step, with a given significance level $\alpha$, a test for equal distributions of $B$ and each $A_{s}$ can be implemented by nonparametric resamples. The energy distance between $A_{s}$ and $B$ is calculated as $e\left(A_{s},B\right)$. Then, $A_{s}$ and $B$ are merged into a pooled set as $U=\left(A_{s},B\right)=\left(\vec{a}_{1},\cdots,\vec{a}_{n_{A_{s}}},\vec{b}_{1},\cdots,\vec{b}_{n_{B}}\right)$. With elements denoted in the same form, this pooled set can be expressed as $U=\left(\vec{u}_{1},\cdots,\vec{u}_{n_{A_{s}}},\vec{u}_{n_{A_{s}}+1},\cdots,\vec{u}_{n_{A_{s}}+n_{B}}\right)$. With the size of $U$expressed as $n_{U}=n_{A_{s}}+n_{B}$, a resample is made without replacement from the pooled set and get a random sample $\left(\vec{u}^{(1)}_{1},\cdots,\vec{u}^{(1)}_{n_{A_{s}}},\vec{u}^{(1)}_{n_{A_{s}}+1},\cdots,\vec{u}^{(1)}_{n_{U}}\right)$. The superscript represents the serial number of this resample. Then, $\left(\vec{u}^{(1)}_{1},\cdots,\vec{u}^{(1)}_{n_{A_{s}}},\vec{u}^{(1)}_{n_{A_{s}}+1},\cdots,\vec{u}^{(1)}_{n_{U}}\right)$ is separated into two subsets as $A_{s}^{(1)}=\left(\vec{u}^{(1)}_{1},\cdots,\vec{u}^{(1)}_{n_{A_{s}}}\right)$ and $B^{(1)}=\left(\vec{u}^{(1)}_{n_{A_{s}}+1},\cdots,\vec{u}^{(1)}_{n_{U}}\right)$. Because $A_{s}^{(1)}$ and $B^{(1)}$ are generate from the first resample of $U=\left(A_{s},B\right)$, the energy distance between two subsets can be calculated and denoted as $e^{(1)}\left(A_{s},B\right)$. In this way, $R$ resamples are made from the pooled set $U$ and obtain energy distances as $\left\{e^{(1)}\left(A_{s},B\right),\cdots,e^{(R)}\left(A_{s},B\right)\right\}$. For $A_{s}$ and $B$, the null hypothesis $H_{0}$ is that the distributions generating them are equal. For the significance level $\alpha$, the null hypothesis would be accepted if $e\left(A_{s},B\right)$ does not exceed $100\times(1-\alpha)\%$ of $\left\{e^{(1)}\left(A_{s},B\right),\cdots,e^{(R)}\left(A_{s},B\right)\right\}$. Put it in another way, a parameter can be estimated for $A_{s}$ and $B$ from $R$ resamples as $e_{accept}\left(A_{s},B,\alpha\right)=(1-\alpha)\times\max\left\{e^{(r)}\left(A_{s},B\right),r=1,\cdots,R\right\}$. For $\left\{A_{s},s=1,\cdots,S\right\}$, the set of parameters $\left\{e_{accept}\left(A_{s},B,\alpha\right),s=1,\cdots,S\right\}$ can be estimated similarly. A common parameter is set for $\left\{A_{s},s=1,\cdots,S\right\}$ and $B$ as $e_{accept}\left(B,\alpha\right)=\min\left\{e_{accept}\left(A_{s},B,\alpha\right),s=1,\cdots,S\right\}$. Then, through additional $R_{1}$ resamples, the likelihood between $B$ and each of $\left\{A_{s},s=1,\cdots,S\right\}$ can be estimated as:

$p\left(A_{s},B,\alpha\right)=\frac{1}{R_{1}}\sum_{r_{1}=1}^{R_{1}} I\left(e^{(r_{1})}\left(A_{s},B,\alpha\right)\leq e_{accept}\left(B,\alpha\right)\right)$ (S35)

where $p\left(A_{s},B,\alpha\right)$ is the likelihood between $B$ and $A_{s}$, $e^{(r_{1})}\left(A_{s},B,\alpha\right)$ is the energy distances from the $r_{1}$th resample of the pooled set $\left(A_{s},B\right)$, $I\left(\cdot\right)$ is the indicator function. If $e^{(r_{1})}\left(A_{s},B,\alpha\right)\leq e_{accept}\left(B,\alpha\right)$, $I\left(e^{(r_{1})}\left(A_{s},B,\alpha\right)\leq e_{accept}\left(B,\alpha\right)\right)=1$. Otherwise, $I\left(e^{(r_{1})}\left(A_{s},B,\alpha\right)\leq e_{accept}\left(B,\alpha\right)\right)=0$. In our simulations, $R_{1}=R$.

In the second step, the identification of $B$ depends on the likelihoods $\left\{p\left(A_{s},B,\alpha\right),s=1,\cdots,S\right\}$. For the significance level $\alpha$, the set $A_{s'}$ is assumed to have the maximum of $\left\{p\left(A_{s},B,\alpha\right),s=1,\cdots,S\right\}$. If $p\left(A_{s'},B,\alpha\right)$ is larger than $1-\alpha$, the distribution generating $B$ is considered to be equal with the distribution generating $A_{s'}$. If there are at least two sets having the largest likelihood, the identification can be made through sampling with the equal probabilities. If the maximum of $\left\{p\left(A_{s},B,\alpha\right),s=1,\cdots,S\right\}$ is not larger than $1-\alpha$, the likelihood between $B$ and each of $\left\{A_{s},s=1,\cdots,S\right\}$ is not large enough. Then, the identification of $B$ can be made through sampling. $p_{s'}$ stands for the probability to accept that the distribution generating $B$ is equal with the distribution generating $A_{s'}$. With likelihoods $\left\{p\left(A_{s},B,\alpha\right),s=1,\cdots,S\right\}$ estimated, $p_{s'}$ can be expressed as:

$p_{s'}=\exp\left(p\left(A_{s'},B,\alpha\right)\right)/\sum_{s} \exp\left(p\left(A_{s},B,\alpha\right)\right).$ (S36)

The identification of $B$ can be made by sampling from the distribution. In this way, not only the likelihoods between $B$ and $\left\{A_{s},s=1,\cdots,S\right\}$ but also the identification of $B$ can be performed in the unsupervised method.

In this paper, the network uses the unsupervised method to identify the outside stimuli. In our simulations, the second-layer excitatory neural spikes perform as the sensory data for identifications. The temporal second-layer excitatory neural spikes represent the current sensory responses induced by the outside stimulus. These induced responses are used to identify the stimuli. As introduced in section of Methods, the previous identified sensory data have been collected into a clustering set for the latter identifications. Each clustering set represents a distribution of the second-layer excitatory sensory responses from the correct identifications to a stimulus. To identify which stimulus inducing the current second-layer responses, the likelihood between the current second-layer responses and each clustering set could be calculated based on the energy distance.

For the non-blank vector of second-layer excitatory spikes $\left(z_{t1},\cdots,z_{tK}\right)$ at time $t$ and the common significance level $\alpha$, the energy distances $\left\{e\left(c_{s},\alpha,t\right),s=1,\cdots,S\right\}$ between second-layer excitatory spikes and clustering sets, as well as the parameters $\left\{e_{accept}\left(c_{s},\alpha,t\right),s=1,\cdots,S\right\}$, could be estimated. $\left\{c_{s},s=1,\cdots,S\right\}$ are the clustering sets denoted. In our simulations, a common received parameter is set for all the clustering sets as $e_{accept}\left(\alpha,t\right)=\min\left\{e_{accept}\left(c_{s},\alpha,t\right),s=1,\cdots,S\right\}$. Then, $\left\{p\left(c_{s},\alpha,t\right)=p\left(e\left(c_{s},\alpha,t\right)\leq e_{accept}\left(\alpha,t\right)\right),s=1,\cdots,S\right\}$ could be estimated for the sets $\left\{c_{s},s=1,\cdots,S\right\}$. With the common $\alpha$ and $e_{accept}\left(\alpha,t\right)$, these probabilities represent the likelihoods between the second-layer excitatory responding vector $\left(z_{t1},\cdots,z_{tK}\right)$ and all the clustering sets.

The temporal action $a_{t}$ performs as the identification of the stimulus at time $t$. If the maximal value of $\left\{p\left(c_{s},\alpha,t\right),s=1,\cdots,S\right\}$ exceeds a predefined value $1-\alpha$, the second-layer excitatory responding vector is considered to be quite similar to the special clustering set with the largest likelihood. If the maximal value of $\left\{p\left(c_{s},\alpha,t\right),s=1,\cdots,S\right\}$ is smaller than $1-\alpha$, the estimation is generated through sampling from the softmax distribution. The softmax distribution of $a_{t}$ to choose the $s'$th clustering set is defined as:

$p\left(a_{t}=s'\right)=\exp\left(p\left(c_{s'},\alpha,t\right)\right)/\sum_{s} \exp\left(p\left(c_{s},\alpha,t\right)\right)$ (S37)

To update clustering sets, the temporal action $a_{t}$ is compared with the stimulus and the temporal reward $r_{t}$ is obtained. If $r_{t}=1$, the corresponding clustering set is updated in a First-In-First-Out (FIFO) manner. In each update, the second-layer excitatory responding vector would be added as the novel component to the end of the clustering set. After adding, if the size of the clustering set is larger than $n_{cluster}$, the redundant components are deleted from the beginning of the set.

Each stimulus is presented to the network for $T_{pre}$ time steps. In each learning simulation, the action and the reward are set to be 0 at the beginning. If the previous reward is 0, it means the previous temporal identification is not correct and the temporal action will be sampled at the novel time step. If the previous reward is 1, it means that the estimation is correct and the temporal action will be hold and not necessary to be resampled. At each time step, the connective weights and clustering sets will be updated according to the temporal reward. At the end of each simulation, each pair of different clustering sets will compare with each other to decide whether to merge or not. In our simulations, $T_{pre}=20\text{ timestep}$, $p_{\max}=0.8$, $n_{cluster}=T_{pre}$, $\alpha=0.05$, $R=50$.

***References***

1. Kappel, D., Nessler, B. & Maass, W. STDP installs in winner-take-all circuits an online approximation to Hidden Markov Model learning. *PLoS Comput Biol* **10**, e1003511 (2014).

2. Jonke, Z., Legenstein, R., Habenschuss, S. & Maass, W. Feedback inhibition shapes emergent computational properties of cortical microcircuit motifs. *J Neurosci* **37**, 8511–8523 (2017).

3. Rueckert, E., Kappel, D., Tanneberg, D., Pecevski, D. & Peters, J. Recurrent spiking networks solve planning tasks. *Sci Rep* **6**, 21142 (2016).

4. Legenstein, R., Jonke, Z., Habenschuss, S. & Maass, W. A probabilistic model for learning in cortical microcircuit motifs with data-based divisive inhibition. *ArXiv* arXiv:1707.05182v1 (2017).

5. Heinerman, J., Haasdijk, E. & Eiben, A. E. Unsupervised identification and recognition of situations for high-dimensional sensori-motor streams. *Neurocomputing* **262**, 90–107 (2017).
